# Herbs and Spices- Biomarkers of Intake Based on Human Intervention Studies – A Systematic Review

**DOI:** 10.1186/s12263-019-0636-8

**Published:** 2019-05-22

**Authors:** Rosa Vázquez-Fresno, Albert Remus R. Rosana, Tanvir Sajed, Tuviere Onookome-Okome, Noah A. Wishart, David S. Wishart

**Affiliations:** 1 Department of Biological Sciences, University of Alberta, Edmonton, AB T6G 2E9 Canada; 2 Department of Computing Science, University of Alberta, Edmonton, AB T6G 2E8 Canada

**Keywords:** Food exposure biomarker, Metabolomics, Herbs, Spices, Diet

## Abstract

Culinary herbs and spices have been used as both food flavoring and food preservative agents for centuries. Moreover, due to their known and presumptive health benefits, herbs and spices have also been used in medical practices since ancient times. Some of the health effects attributed to herbs and spices include antioxidant, anti-microbial, and anti-inflammatory effects as well as potential protection against cardiovascular disease, neurodegeneration, type 2 diabetes, and cancer. While interest in herbs and spices as medicinal agents remains high and their use in foods continues to grow, there have been remarkably few studies that have attempted to track the dietary intake of herbs and spices and even fewer that have tried to find potential biomarkers of food intake (BFIs). The aim of the present review is to systematically survey the global literature on herbs and spices in an effort to identify and evaluate specific intake biomarkers for a representative set of common herbs and spices in humans. A total of 25 herbs and spices were initially chosen, including anise, basil, black pepper, caraway, chili pepper, cinnamon, clove, cumin, curcumin, dill, fennel, fenugreek, ginger, lemongrass, marjoram, nutmeg, oregano, parsley, peppermint and spearmint, rosemary, saffron, sage, tarragon, and thyme. However, only 17 of these herbs and spices had published, peer-reviewed studies describing potential biomarkers of intake. In many studies, the herb or spice of interest was administrated in the form of a capsule or extract and very few studies were performed with actual foods. A systematic assessment of the candidate biomarkers was also performed. Given the limitations in the experimental designs for many of the published studies, further work is needed to better evaluate the identified set of BFIs. Although the daily intake of herbs and spices is very low compared to most other foods, this important set of food seasoning agents should not be underestimated, especially given their potential benefits to human health.

**Electronic supplementary material:**

The online version of this article (10.1186/s12263-019-0636-8) contains supplementary material, which is available to authorized users.

## Background

Spices are the dried, pleasantly aromatic parts of the plants. More specifically, as defined by the Food and Drug Administration organization (FDA), spices are: “aromatic vegetable substances, in the whole, broken, or ground form, whose significant function in food is seasoning rather than nutrition” [1]. The main difference between a herb and a spice is that a spice comes from any part of a plant other than the leaves while a herb always comes from the leaves [1]. Spices typically come from the dried part of a plant such as buds, flowers (cloves, saffron); bark (cinnamon); root (ginger, turmeric); fruits/berries (cloves, chili, black pepper); or seeds (cumin) that contain volatile oils or aromatic scents and flavors [1, 2] (see Table 1). Most of the known herbs and spices originate from Mediterranean countries, the Middle East or Asia, and many have been used since ancient Egyptian and Roman times [3].

**Table 1:** Scientific and common names of selected spices evaluated in this review

Num.	Name of spice	Scientific name	Part of the plant
1	Anise	*Pimpinella anisum*	Seed/fruit
2	Basil	*Ocimum basilicum*	Leaf
3	Black pepper	*Piper nigrum*	Berry
4	Caraway	*Carum carvi*	Fruit
5	Chili pepper	*Capsicum annuum, Capsicum baccatum, Capsicum chinense, Capsicum frutescens, Capsicum pubescens*	Fruit
6	Cinnamon	*Cinnamomum*	Bark
7	Clove	*Syzygium aromaticum*	Bud
8	Cumin	*Cuminum cyminum*	Seed
9	Dill	*Anethum graveolens*	Leaf/seed
10	Fennel	*Foeniculum vulgare*	Leaf/seed
11	Fenugreek	*Trigonella foenum-graecum*	Seed
12	Ginger	*Zingiber officinale*	Root
13	Lemongrass	*Cymbopogon*	Leaf
14	Marjoram	*Origanum majorana*	Leaf
15	Nutmeg	*Myristica fragrans*	Seed
16	Oregano	*Origanum vulgare*	Leaf
17	Parsley	*Petroselinum crispum*	Leaf
18	Peppermint	*Mentha x piperita*	Leaf
19	Rosemary	*Rosmarinus officinalis*	Leaf
20	Saffron	*Crocus sativus*	Stigma (flower)
21	Sage	*Salvia officinalis*	Leaf
22	Spearmint	*Mentha spicata*	Leaf
23	Tarragon	*Artemisia dracunculus*	Leaf
24	Thyme	*Thymus vulgaris*	Leaf
25	Turmeric (curcumin)	*Curcuma longa*	Root

Herbs and spices have played, and continue to play, important roles as flavoring agents, food preservatives and medicines for centuries. Over the last few decades, research into their health benefits has increased significantly, as many herbs and spices are known to possess properties associated with reducing the risk of developing chronic diseases. In particular, some of the potential health benefits of herbs and spices include conferring protection against cardiovascular disease, neurodegenerative conditions, chronic inflammation, cancer, obesity, and type 2 diabetes [4–14]. A number of herbs and spices have also been noted for their strong antioxidant, anti-microbial, and anti-inflammatory properties [4, 7, 15]. Moreover, the flavoring properties of many herbs and spices tend to reduce the use of salt as a flavoring agent (i.e., reduced sodium intake) which has additional cardiovascular health benefits [16].

Most of the positive health effects of herbs and spices towards preventing or ameliorating chronic diseases such as cancer, cardiovascular disease, arthritis, and neurodegeneration appear to be mediated through the direct action of their constituent phytochemicals (particularly polyphenols or polyphenol breakdown products) targeting specific receptors or enzymes involved in various anti-inflammatory pathways or immune responses [10]. Herbs and spices (especially in their dried form) contain high levels of polyphenols [6] and other physiologically active phytochemicals. The predominant class of polyphenols found in herbs and spices are the phenolic acids and flavonoids (mainly flavones and flavonols) [17]. Relative to other polyphenol-rich foods such as broccoli, dark chocolate, red, blue and purple berries, grapes or onions—herbs and spices generally contain somewhat higher levels of these compounds. For instance, oregano has 935.3 mg of total phenolic content per 100 g of fresh weight (FW) in the fresh form (F) while in the dried form (D) has 6367 mg/100 g. Similarly, high polyphenolic levels are seen in rosemary [1082.4 mg/100 g (F) vs. 2518 mg/100 g (D)], thyme [1173.28 mg/100 g (F) vs. 1815 mg/100 g (D)], and parsley [89.27 mg/100 g (F) vs. 1584 mg/100 g (D)]. Likewise, cloves have 16,047.25 mg/100 g, cinnamon 9700 mg/100 g, and turmeric 2117 mg/100 g (all FW). In contrast, other non-herbs and foods such as dark chocolate contain 1859.8 mg/100 g FW, while raw blackcurrants contain 820.6 mg/100 g FW and broccoli just 198.6 mg/100 g FW.

Polyphenols, terpenoids, and other spice-derived alkaloids (such as capsaicinoids) are also known to possess antibacterial, antiviral, and antifungal properties [18]. This is one reason why herbs and spices are so frequently used as preservative agents in food [19]. The antimicrobial properties of herbs and spices have been attributed to their unique volatile oils and oleoresins [20]. For instance, comparative studies involving cloves, cinnamon, oregano, rosemary, sage, and thyme showed that thyme oil was particularly active against *Aeromonas hydrophila—*a pathogen widely distributed in the environment, domestic animals, and food [21]. Likewise, essential oils found in thyme, oregano, mint, cinnamon, and cloves were found to possess strong antibacterial properties against several food-borne bacteria and fungi [22].

The number of herbs with known or potential anti-inflammatory activity is quite significant. Of the 25 herbs and spices analyzed in this review, 21/25 (84%) had at least one published study supporting an anti-inflammatory finding. The spices that are most frequently identified as having anti-inflammatory effects are thyme, oregano, rosemary, sage, basil, mint, turmeric, dill, parsley, cinnamon, clove, nutmeg, lemon grass, ginger, chili pepper, fenugreek, and pepper [4, 23]. Many of the anti-inflammatory compounds found in herbs and spices, such as curcumin, gingerol, and capsaicin, appear to operate by inhibiting one or more of the steps linking pro-inflammatory stimuli with cyclooxygenase (COX) activation [10]. While the mechanisms behind some of the health benefits in herbs and spices are becoming clearer over time, the vast majority of herbs and spices still have rather ill-defined health benefits and yet-to-be-identified chemical “actors” [4].

Given the widespread use of herbs and spices and given their known (and potential) health benefits, there is clearly a need to better understand the consumption patterns of herbs and spices. Population-wide average dietary intake of common spices varies considerably around the world. For instance, Europeans consume an estimated at 0.5 g/person per day, Australians and New Zealanders consume between 1.3–1.9 g/day, and residents of Africa consume 1.8 g/day. Moderate consumers of herbs and spices are found in the Middle East and Eastern Asia with daily consumption of 2.6 and 3.1 g/person, respectively. The highest consumers of herbs and spices are found in India, South Africa, and Latin America with an average of 4.4 g/day [23, 24]. In India, turmeric consumption, alone, has been estimated to be 1.5 g/person per day [25]. While consumption of herbs and spices is generally higher in Southern countries such as India, Mexico, Peru, China, and Thailand, herb and spice intake has been increasing in many developed countries in Northern Europe and North America, due to changing food habits and a growing preference for ethnic or spicy food [26, 27].

While broad estimates of herb and spice consumption are useful, more detailed information of what herbs/spices and how much of each are being consumed would be much more useful. In this regard, the development and identification of biomarkers of food intake (BFI) for specific herb and spice consumption would help advance the field. In particular, herb-specific or spice-specific BFIs would permit better exposure estimates for much more comprehensive and far more detailed epidemiological studies of the influence of herbs and spices on human health. This review is focused on finding and evaluating specific nutritional biomarkers for a representative set of 25 common herbs and spices used worldwide [3, 10].

## Methods

### Selection of herbs and spices

This set of 25 was selected based on reported estimates of consumption volume in North America and Europe as well as the frequency with which these spices and herbs were cited in the literature. The 25 herbs and spices that were examined include anise, basil, black pepper, caraway, *Capsicum* sp. (chili pepper and paprika), cinnamon, clove, cumin, curcumin, dill, fennel, fenugreek, ginger, lemongrass, marjoram, nutmeg, oregano, parsley, peppermint and spearmint, rosemary, saffron, sage, tarragon, and thyme (Table 1).

### Primary literature search

A systematic BFI review should consist of an extensive literature search (ELS) for each food or food group, which will select from all the putative markers proposed in the available scientific literature only the most promising candidate biomarkers based on their likely specific presence in the food and/or food group [28]. The structure of the present guidelines for conducting an ELS on putative and candidate BFIs is reported elsewhere [29], which follows the methods proposed by the European Food Safety Authority (EFSA) for conducting systematic reviews for food and feed safety assessments [30], as well as the ‘Cochrane handbook for systematic review on interventions’ [31], with proper modifications for handling BFIs. The PRISMA statement for reporting and discussing the results [32] was also used. Original research papers and reviews were searched in three databases: PubMed, Scopus, and the ISI Web of Knowledge using combinations of the grouped search terms (biomarker* OR marker* OR metabolite* OR biokinetics OR biotransformation) AND (trial OR experiment OR study OR intervention) AND (human* OR men OR women OR patient* OR volunteer* OR participant*) AND (urine OR plasma OR serum OR blood OR excretion) AND (intake OR meal OR diet OR ingestion OR consumption OR eating OR drink* OR administration) as reported in in Additional file 1: Table S1, together with specific keywords related to herbs and spices food group (Additional file 1:Table S2)*.* The specific search terms for specific herbs and spices included both their common and scientific names so as to be as comprehensive as possible*.* The specific keywords for the herbs and spices of interest were the following: *(“anise”* OR *“Pimpinella anisum”* OR *“basil”* OR *“Ocimum basilicum”* OR *“black pepper”* OR *“Piper nigrum”* OR *“caraway”* OR *“Carum carvi”* OR *“chili pepper”* OR *“Capsicum annuum”* OR *“Capsicum baccatum”* OR *“Capsicum chinense”* OR *“Capsicum frutescens”* OR *“Capsicum pubescens”* OR *“cinnamon”* OR *“Cinnamomum”* OR *“clove”* OR *“Syzygium aromaticum”* OR *“cumin”* OR *“Cuminum cyminum”* OR *“turmeric”* OR *“Curcuma longa”* OR *“dill”* OR *“Anethum graveolens”* OR *“fennel”* OR *“Foeniculum vulgare”* OR *“fenugreek” OR “Trigonella foenum-graecum”* OR *“ginger”* OR *“Zingiber officinale”* OR *“lemongrass”* OR *“Cymbopogon”* OR *“marjoram”* OR *“Origanum majorana”* OR *“nutmeg”* OR *“Myristica fragrans”* OR *“oregano”* OR *“Origanum vulgare”* OR *“parsley”* OR *“Petroselinum crispum”* OR *“peppermint”* OR *“Mentha x piperita”* OR *“rosemary”* OR *“Rosmarinus officinalis”* OR *“saffron”* OR *“Crocus sativus”* OR *“sage”* OR *“Salvia officinalis”* OR *“spearmint”* OR *“Mentha spicata”* OR *“tarragon”* OR *“Artemisia dracunculus”* OR *“thyme”* OR *“Thymus vulgaris”.* To improve the accuracy of the search and to help exclude unrelated papers, several other keywords were used including NOT (Ginger [Author] OR Parsley [Author] OR Sage [Author] OR Dill [Author] OR Pimpinella [Author] OR Basil [Author] OR Artemisia [Author] OR Cumin [Author] OR Thyme [Author]). The default search fields for each of the databases were [All Fields] for PubMed, [Article Title/ Abstract/ Keywords] for Scopus, and [Topic] for ISI Web of Science, respectively. This literature search was conducted between November 2015 and January of 2016, followed by a second validation phase and update in January 2018. The literature search was limited to papers in the English language with no restrictions being applied for the publication dates. Results of this literature search for potential biomarkers of herbs and spices are shown in Table 2.

**Table 2:** List of studies reporting candidate biomarkers for herb and spice intake

Dietary factor	Study design	Subjects	Analytical method	Sample type	Discriminating metabolites/candidate biomarkers	Primary Ref.
Anise (anethole admn.)	Acute human study. [methoxy-^14^C]-labeled compound	5 (males)	Radiochemical (^14^C labeled) and HPLC	Urine (2 h–10 h, 24 h, and 48 h)	4-methoxybenzoic acid, 4-methoxyhippuric acid, 3 unknown compounds	[36]
Anise-based alcoholic drink	Dose escalating study (120 ml, 200 ml, 360 ml “Helenas Ouzo” (anethole-containing drink)	1	HS-SPME-GC–MS	Serum (1, 2, 4, 8, and 24 h)	Anethole	[37]
	Observational study: drivers under the influence of alcoholic-containing anethole drink	50		Serum		
*Capsicum* sp. Chili pepper (capsule)	Acute crossover study (5 g of capsicum extract)	12 (males)	HPLC	Plasma	Capsaicin	[41]
*Capsicum* sp. CH-19 sweet non-pungent red pepper (capsule)	Double-blind, randomized, placebo-controlled, dose-escalating (15 or 30 mg capsinoids extract)	24 (males)	LC-MS/MS and HPLC-UV	Plasma (15 min, 30 min, 1 h, 2 h, 4 h, 8 h, and 24 h)	Capsiate, dihydrocapsiate, nordihydrocapsiate, vanillyl alcohol	[150]
*Capsicum* sp. Paprika carotenoids	Case study. 200 ml paprika carotenoid beverage	5 (young, healthy)	HPLC-UV-VIS and Q-TOF-MS/MS	Plasma (0 week, 2 weeks, 4 weeks), erythrocytes	β-cryptoxanthin, cucurbitaxanthin A, cryptocapsin, lutein, zeaxanthin, capsanthin, capsanthone	[42]
Cinnamon	Four-way crossover study	24	HPLC MS/MS	Plasma and urine	7-hydroxycoumarin	[48]
Fennel (fennel tea)	Single-dose acute study (500 ml of fennel tea)	7	LC-MS/MS and GC-GC-MS	Urine (1.5, 4, 8, 14, 24 h)	Estragole, 1′-hydroxyestragole, *trans*-Anethole, -Allylphenol-G	[63]
	Dose-escalation study (250, 500, 1000 ml fennel tea).	1		Plasma (0.75,1.5, 2, 2.5 h)		
Fennel, basil, and tarragon	15 mL fennel extract; 15 ml tarragon extract; 15 ml basil brewed	NP	IS-R-DLLME and HPLC	Plasma (2 h, 4 h, and 8 h) and Urine (3 h, 6 h, and 9 h)	*Para*-anisaldehyde *trans*-anethole estragole	[38]
Ginger (extract)	Acute single dose: 2 g ginger extracts	9 (healthy)	LC-MS/MS	Plasma (0.25 h, 0.5 h, 0.75 h, 1 h, 2 h, 4 h, 6 h, 10 h, 24 h, 48 h, and 72 h)	10-Gingerol, 6-Shogaol, 6-Gingerol-G, 8-Gingerol-G 10-Gingerol-G, 6-Shogaol-G 6-Gingerol-S, 8-Gingerol-S, 10-Gingerol-S, 6-Shogaol-S	[70]
	Multiple dose: 24-day randomized controlled trial. 250 mg ginger extract	30 (healthy)		Plasma (0–24 h) and colon (biopsy)	6-Gingerol-G (plasma), 10-Gingerol-G (plasma), 6-Gingerol-S (plasma), 10-Gingerol-G (colon), 10-Gingerol-S (colon)	
	Multiple dose: 24-day randomized controlled trial. 250 mg ginger extract	20 (high-risk colorectal cancer)		Plasma (0-24 h) and colon (biopsy)	6-Gingerol-G (plasma), 10-Gingerol-G (plasma), 6-Gingerol-S (plasma) 10-Gingerol-G (colon), 10-Gingerol-S (colon)	
Ginger	Dose escalation study: 100 mg, 250 mg, 500 mg, 1 g, 1.5 g, 2 g ginger extract (capsule)	27 (healthy)	HPLC-ECD, HPLC-UV	Plasma (15 min, 30 min, and 45 min, 1 h, 2 h, 4 h, 6 h, 10 h, 24 h, 48 h, and 72 h)	6-Gingerol-G, 8-Gingerol-G, 10-Gingerol-G, 6-Shogaol-G, 6-Gingerol-S, 10-Gingerol-S	[69]
Ginger (ginger tea)	Acute study. 2× (18 g/bag) ginger tea *(*focused on the metabolism of shogaol)	3 (healthy males)	LC/ESI-MS/MS	Urine (0–2 h, 2–4 h, 4–6 h, 6–9 h, 9–12 h, and 12–24 h)	5-Cys-6S, 5-NAC-6S, 5-Cys-Gly-6S, 5-Cys-M6, 5-NAC-M6, 5-Cys-Gly-M6, 5-Cys-8S, 5-Cys-M6’, 5-Cys-10S, 5-Cys-M6"	[71]
Marjoram (extract)	Acute single oral dose (3.75 g) of *O. onites* extract	6 (healthy)	HPLC-CEAD	Urine (24 h, 48 h)	Protocatechuic acid, *p*-hydroxybenzoic acid, caffeic acid, ferulic acid, syringic acid, vanillic acid, *p*-coumaric acid, 3,4-dihydroxyphenylacetic acid, *m*-hydroxyphenylacetic acid	[97]
Nutmeg	Acute oral dose in rats (100 mg/kg body mass) of EL, MY, and SA or a single 500 mg/kg body mass of nutmegs	2 rats × each substance and dose	GC-MS	Urine (24 h)	O-demethyl elemicin^*^, O-demethyl dihydroxy elemicin^*^, demethylenyl myristicin^*^, dihydroxy myristicin^*^, demethylenyl safrole^*^	[73]
	Observational exploratory toxicological study: after nutmeg abuse (~ 5 nutmegs)	1				
Oregano (extract)	Oregano extract (25, 75, or 225 mg/kg	15 mice	HPLC–MS/MS	Plasma and brain tissue	Carvacrol	[95]
Parsley	Randomized crossover with two 1-week intervention periods in succession, supplemented with parsley 20 g parsley/MJ	14 (healthy)	HPLC-DAD	Urine (24 h)	Apigenin	[108, 109]
Parsley	Acute human study. (149.45 ± 35.21 g parsley)	11 (healthy)	HPLC-ECD	Plasma (4–11 h, 28 h), urine (24 h), and red blood cells	Apigenin	[102]
Peppermint oil (capsule)	Acute pharmacokinetic study. Intake of 0.4 ml peppermint oil in either colpermin or gelatine capsules (91–97 mg capsule)	6 (healthy)	NP	Urine (24 h)	Menthol-G	[117]
		6 (ileostomy)				
Peppermint oil	Acute randomized intake of 0.6 ml peppermint oil in either *Colpermin* or *Mintec* preparations	13 (healthy)	GC-MS	Urine (2 h-interval for 14 h + single overnight (10 h)	Menthol-G	[116]
Peppermint oil (capsule)	180 mg peppermint oil enteric-coated capsule (peroral administration)	4 (males)	GC-FID	Urine (2-h interval up to 14 h)	Menthol-G	[118]
Peppermint oil (capsule)	Acute (400 mg peppermint oil in enteric-coated capsule) and repeated 4 weeks later	5 (healthy)	^2^H-NMR	Urine (2 h, 4 h, 6 h, and 8 h)	Menthol-G	[120]
Peppermint oil (capsule)	(1) 400 mg of enteric-coated peppermint oil capsules and 6 g of 99% [U-^13^C] glucose	1 (female)	^13^C-NMR	Urine (2-4 h)	^13^C-menthol-G	[119]
	(2) Primed infusion of [U-^13^C] glucose + 400 mg enteric-coated peppermint oil capsules	4 (severe heart failure)		Urine (2 h)		
Peppermint oil (L-menthol preparation)	Escalating-single-dose, randomized, double-blind, placebo-controlled (menthol preparation, 80–320 mg). Intragastric spraying of peppermint oil	24 (males)	GC-MS	Plasma (5, 10, 30, 60, 120, and 240 min and 8, 12, and 24 h after each dose)	Menthol, menthol-G, M7, M9, M11, M29	[121]
				Urine (before dosing (−12–0 h) and 0–4 h, 4–8 h, 8–12 h, and 12–24 h after	Menthol-G, M2, M3–11, M12, M13–18, M19–21, M22–28, M29, M30–32.	
Rosemary (extract)	Acute, controlled, randomized study. Rosemary extract enriched in carnosic acid 40% (*w*/*w*)	24 Zucker rats	HPLC/QTOF-MS and HPLC-UV	Gut, liver, plasma, brain,	Carnosic acid-G, carnosol-G, rosmanol-G, carnosic acid 12 methyl ether, 5,6,7,10-tetrahydro-7-hydroxyrosmariquinone, carnosic glutathione oxidized, carnosol-S, rosmanol-S, rosmarinic acid, carnosic cysteine, carnosic glutathione, rosmadial-G, rosmanol, ipirosmanol, epiisorosmanol, rosmadial/rosmanol quinone, rosmanol/epirosmanol methyl ether, carnosol, rosmadial methyl ether, epirosmanol ethyl ether, epiisorosmanol methyl ether, carnosol methyl ether, carnosic acid.	[100]
	Subchronic, controlled, randomized study Rosemary extract enriched in carnosic acid 40% (*w*/*w*) (64 days)					
Saffron (tea)	Single-dose acute study. 200 mg saffron in 150 ml water (saffron tea)	4 (healthy)	SPE-HPLC-DAD	Plasma (0 h, 2 h, and 24 h)	*cis-*Crocetin, *trans-*Crocetin	[126]
Saffron (purified crocetin)	Open-label, single dose escalation of crocetin (7.5, 15 and 22.5 mg)	10 (healthy)	HPLC	Plasma (1 , 2 h, 4 h, 6 h, 8 h, 10 h, and 24 h)	Crocetin	[127]
Sage (tea)	Acute human study (1.02 mg 1,8-cineole) in sage tea	1 (female)	SPME-GC-MS and LC-MS/MS	Plasma (0.75 h, 1.7 h, 3.25 h, 6.75 h, and 24 h) and urine (2 h, 5 h, 7 h, 10 h, 17 h, 21 h, 28 h, 32 h, 35 h, 44 h, 50 h, 53 h, 60 h, and 69 h)	1,8-cineole, 2-hydroxy-1,8-cineole, 3-hydroxy-1,8-cineole, 7-hydroxy-1,8-cineole, 9-hydroxy-1,8-cineole.	[130]
Thyme (tablet)	Acute study. A single dose of a Bronchipret® TP (tablet equivalent to 1.08 mg thymol)	12	HS-SPME-GC-MS and LC-MS/MS	Plasma (0.25 h, 0.5 h, 0.75 h, 1 h, 1.5 h, 2 h, 2.5 h, 3 h, 3.5 h, 4 h, 5 h, 6 h, 7 h, 8 h, 9 h, 10 h, 11 h, 12 h, 14 h, 24 h, 31 h, 38 h, 48 h, 55 h, 62 h, and 72 h)	Thymol-S	[151]
				Urine (0 to 3 h, 3 to 6 h, 6 to 9 h, 9 to 14 h, 14 to 24 h, 24 to 31 h, 38 to 48 h, 48 to 55 , 55 to 62 h, and 62 to 72 h)	Thymol-G, thymol-S	
Thyme	Acute intake of 1.5 g of thyme extract	12 Wistar rats	μSPE-UPLC-MS/MS	Plasma	Thymol-S, thymol-G, luteolin-S, luteolin-G, hydroxyphenylpropionic acid-S, coumaric acid-S, caffeic acid-S, ferulic acid-S, ferulic acid-G, hydroxybenzoic acid, and dihydrophenylpropionic acid-S	[98]
Thyme (olive oil enriched with thyme polyphenols)	Randomized, double-blind, controlled, cross-over trial. Administration of 25 ml/day (VOO)/VOO + PC/VOO + PC + PC of thyme	33 (hypercholesterolemic)	μSPE-UPLC-ESI-MS/MS	Plasma	Thymol-S, hydroxyphenylpropionic acid-S, caffeic acid-S	[99]
				Urine (24 h)	Thymol-S, Thymol-G, hydroxyphenylpropionic acid-S, *p*-cymene-diol-G, caffeic acid-S	
Thyme (olive oil enriched with thyme)	(1) In vitro colonic fermentation (0 to 48 h)	3 (healthy)	UPLC-ESI-MS/MS and GC-FID	Feces (in vitro fermentation)	Thymol, carvacrol, 2-(3′,4′-dihydroxyphenyl) acetic acid, 2-(4′-hydroxyphenyl) acetic acid, phenylacetic acid, 3-(4′-hydroxyphenyl) propionic acid, phenylpropionic acid.	[96]
					2-(3′,4′-dihydroxyphenyl) acetic acid, 2-(4′-hydroxyphenyl) acetic acid, Phenylacetic acid, 3-(4′-hydroxyphenyl) propionic acid, phenylpropionic acid	
					Caffeic acid, *p*-coumaric acid, 3-(3′, 4′-dihydroxyphenyl) propionic acid; hydroxyphenylpropionic acid; phenylpropionic acid, 2-(3′,4′-dihydroxyphenyl) acetic acid; 2-(4′-hydroxyphenyl) acetic acid; phenylacetic acid	
					3-(3′, 4′-dihydroxyphenyl) propionic acid; hydroxyphenylpropionic acid; phenylpropionic acid, 2-(3′,4′-dihydroxyphenyl) acetic acid, 2-(4′-hydroxyphenyl) acetic acid; phenylacetic acid	
	(2) Human intervention study: 25 ml/day of a thyme phenol-enriched olive oil for 3 weeks	10		Feces (in vivo*,* (0-3wk)	Carvacrol, 2-(4-hydroxyphenyl) acetic acid, 3-(3′-4′-dihydroxyphenyl) propionic acid, hydroxyphenylpropionic acid, phenylpropionic acid	
Turmeric (curcuminoids in capsule)	Randomized double blind placebo (1 g/day, 4 g/day, placebo), 6 months	31 (elderly)	LC-MS/MS	Plasma (2–2.5 h after 1 month)	Curcumin, DMC BDMC, THC, ferulic acid, vanillic acid	[132]
Turmeric (curcuminoids in capsule)	Acute study	2 (healthy)	LC-MS/MS	Plasma	COG	[141]
Turmeric (curcuminoidsin nanoemulsion)	Acute study (2 g nanoemulsion curcuminoids)	2 (healthy)	LC-MS/MS	Plasma	Curcumin, COG, COS, DMC, BDMC, and THC	[142]
Turmeric (curcuminoids in capsule)	Nonrandomized, open-label, phase II trial (starting dose 8 g curcuminoids) 8 weeks	25 (pancreatic cancer)	LC-MS	Plasma (1 h, 2 h, 6 h, 24 h, 48 h, 72 h, day 8 and after 4 weeks	COG and COS	[140]
Turmeric (curcuminoids in capsule)	Dose escalation. 450–3600 mg/day 1 week	12 (hepatic metastasis from colorectal cancer)	HPLC-UV, LC-MS	Plasma and liver tissue	Hexahydrocurcumin (liver), hexahydrocurcuminol (liver), curcumin (plasma), COG (plasma), COS (plasma).	[143]
Turmeric (curcuminoids in capsule)	Acute study.	12 (colorectal carcinoma)	HPLC-UV HPLC-MS	Plasma and colorectal tissue	Curcumin (plasma and colorectal tissue), COG and COS (colorectal tissue)	[6]
Turmeric (curcuminoids-different administration types)	Randomized double blind crossover study with formulated (CP, CTR, CHC) and unformulated (CS) curcumin	12 (healthy)	LC-MS/MS	Plasma (1 h, 2 h, 3 h, 4 h, 5 h, 6 h, 8 h, and 12 h)	Curcumin, DMC, BDMC, THC	[138]
Turmeric (curcuminoids in capsule)	14-day intervention (2.35 g capsule)	24 (colorectal cancer)	UPLC-UV, LC-MS/MS	Plasma, urine and colon tissue	Curcumin, BDMC, DMC, BDMC-S, DMC-S, COS, COG, BDMC-G, DMC-G	[147]
Turmeric (Theracurmin®)	Acute dose escalation 150 mg and 210 mg	6 (healthy)	LC-MS/MS	Plasma (0 h, 1 h, 2 , 4 h, 6 , 24 h)	Curcumin	[152]
Turmeric (Theracurmin®)	Multi-week dose escalation	16 (pancreatic or biliary tract cancer)	LC-MS/MS	Plasma (2 h)	Curcumin	[153]
Turmeric (turmeric fresh derived curcuminoids vs. std. curcumin)	Multi-week double crossover study. 250 mg/kg body weight	18 (mice)	LC-DAD-ESI-MS/MS	Plasma (0 h, 0.5 , 1 h, 3 h, 5 h, 8 h, 12 h)	Curcumin, DMC, BMDC	[149]
	Acute, single-blind crossover study, 100 mg, 250 mg, 1000 mg	15 (healthy)				
Turmeric (C3 complex)	Acute study. 1 single dose (4 g)	8 (healthy)	HPLC	Serum	Curcumin	[154]
	3–4-week intervention study. (8 g/day)	15 (with HNSCC)				
Turmeric (curcuma extract capsule)	Dose escalation: 440 mg–2200 mg/day. 4 months	15 (colorectal cancer)	HPLC-UV	Blood, urine, feces	COS (only detected in feces)	[25]
Turmeric (C3 complex)	Dose escalation study. 4-month intervention (450, 900, 1800, 3600 mg). 4 months	15 (colorectal adenocarcinoma patients)	LC-MS	Plasma, urine, feces	Curcumin (plasma, urine, feces), COG (plasma, urine), DMC (plasma, urine), BDMC (plasma), DMC-G (plasma, urine), DMC-S (plasma) COS (plasma, urine, and feces).	[145]
Turmeric (C3 complex, 10 or 12 g)	Acute study	12 (healthy)	HPLC	Plasma	COG and COS	[139]
Turmeric	3-month intervention with different doses each group (500, 1000, 2000, 4000, 8000 mg/day)	25 cancer	HPLC-UV	Serum (0 h, 0.5 h, 1 h, 1.5 h, 2 h, 2.5 h, 3 h, 4 h, 6 h, 8 h, 12 h, 14 h, and 24 h)	Curcumin (only in serum)	[144]
				Urine (0–2 h, 2–4 h, 4–8 h, and 8–24 h)		
Turmeric (Theracurmin®)	Acute study. Curcumin in powder and Theracurmin® in liquid (30 mg).	12 Sprague-Dawley rats and 14 humans	LC-MS/MS	Plasma	Theracurmin and curcumin	[148]
Turmeric (curcumin)	Dose escalation study. C3 complex adm. 500 mg, 1000 mg, 2000 mg, 4000 mg, 6000 mg, 8000 mg, 10,000 mg, and 12,000 mg)	24	HPLC	Plasma and serum	Curcumin (just in serum at 10000 and 12,000 mg)	[146]

*Adm*, administration; *BDMC*, bisdemethoxycurcumin, *CEAD*, coulometric electrode array detector; *CHC***,** combination of hydrophilic carrier, cellulosic derivatives, and natural antioxidants; ^*13*^*C-NMR***,** carbon nuclear magnetic resonance; *COG*, curcumin-O-glucoronide. *COS*, curcumin-O-sulfate; *CP*, curcumin phytosome formulation; *CS*, standardized curcumin mixture; *CTR* formulation with volatile oils of turmeric rhizome, *Cys*, cysteinyl; *DAD*, diode array detector; *DMC*, demethoxycurcumin, *ECD*, electrochemical detection. *EL*, elemicin; *ESI***,** electrospray ionization; *FID***,** flame ionization detector; *−G*, glucuronide; *Gly*, glycinyl; *H-NMR*, proton nuclear magnetic resonance; *HPLC*, high-performance liquid chromatography; *HNSCC* head and neck squamous cell carcinomas; *HS-SPME*, headspace solid-phase microextraction; *IS-R-DLLME*, in-syringe reversed dispersive liquid-liquid microextraction; *LC*, liquid chromatography; *months*, months; *MJ*, megajoules; *MY*, myristicin, *M6***,** 1-(4′-hydroxy-3′-methoxyphenyl)-4-decen-3-ol; *M6′*, 1-(4′-hydroxy-3′-methoxyphenyl)-4-dodecen-3-ol; *M6′′*, 1-(4′-hydroxy-3′-methoxyphenyl)-4-tetradecen-3-ol; *M29*, menthol sulfoconjugate; *M7, 9, 11*, hydroxyl menthol glucuronide. *M3–11*, hydroxyl menthol glucuronide; *M19–21*, dihydroxyl menthol glucuronide. *M2*, aldehyde-menthol glucuronide; *M13–18*, carboxylate-menthol or aldehyde-hydroxyl menthol glucuronide; *M29–32*, sulfate conjugates; *M12*, dialdehydementhol glucuronide (M12); *MS* mass spectrometry; *NAC***,** N-acetylcysteinyl; *NP*, not provided; *PC*, phenolic compounds; *Q-TOF*, quadrupole time-of-flight; *S*, sulfate; *SA*, safrole; *THC*, tetrahydrocurcumin; *UPLC*, ultra-high performance liquid chromatography; *UV* ultraviolet; *VIS*, visible; *VOO*, virgin olive oil. weeks, week; *w/w*, weight per weight; *μSPE*, microelution solid-phase extraction; *6S*, 6-shogaol; *8S*, 8-shogaol; *10S*, 10-shogaol

*In the study performed in rats, there were other metabolites also identified but not found in the human sample analyzed so they were not considered in this table

### Exclusion criteria

Papers were excluded if they investigated the effect on human physiology of the selected herbs and spices, the presence or effect of toxicants, if they referred to unspecific markers or if they were based on in vitro or animal studies.

### BFI identification and classification

A second search step was used to evaluate the apparent specificity of the markers in the list. The remaining list of potential biomarkers was used for a second literature search in the three bibliographic databases used also for the primary search. This was done in order to identify other foods containing the potential biomarkers or their precursors, as well as foods otherwise associated with these compounds. For the second web-based literature search, the “marker name” was used as keyword, together with AND (biomarker* OR marker* OR metabolite* OR *biokinetics* OR *biotransformation).* Further filters, such as *(urine* OR *plasma* OR *serum* OR *blood* OR *excretion)* AND *(intake* OR *meal* OR *diet* OR *ingestion* OR *consumption* OR *eating* OR drink* OR *administration)* AND (human* OR *men* OR *women* OR patient* OR volunteer* OR participant* OR subject*) were added based on the results obtained. At the end of this selection process, the usefulness, and weakness of each biomarker compounds were evaluated, and the most promising biomarkers were scored to assess their validity as BFIs according to the system reported below.

### Marker validation score

In order to further assess the validity of the biomarker candidates, a set of consensus evaluation criteria, from the FoodBAll consortium was employed [33]. Specifically, the suitability of each biomarker was assessed by answering a set of questions reported elsewhere [33], which reflect the analytical and biological criteria that the proposed biomarkers should fulfill in order to be considered valid. Such questions have been answered for the most promising biomarkers and the results are reported in Table 3. Possible answers were Y (yes), N (no), or U (unknown or uncertain). The potential markers were scored for plausibility and uniqueness (question 1); kinetics and dose-response relationship (question 2), kinetics of postprandial response (question 3a) and longer-term kinetics (question 3b). All markers were further evaluated for their robustness in complex diets or a real exposure situation (question 4) and reliability (question 5), which refers to the concordance with other measures of intake for the food or food group in question (such as other existing validated biomarkers or dietary instruments). The analytical aspects of each BFI were investigated through an evaluation of their chemical stability (question 6), their analytical performance (question 7), and reproducibility in different labs (question 8).

**Table 3:** Summary of the selected candidate BFIs of herbs and spices and their assessment relative to the nine validation criteria/questions described in [33]

Food item	Metabolites	Biofluid locations	Questions^*^ 1 2 3a 3b 4 5 6 7 8								
Anise	Anethole	Serum	N	Y	Y	U	U	Y	U	Y	N
	4-methoxyhippuric acid^1^	Urine	N	Y	Y	U	U	U	Y	N	N
	4-methoxybenzoic acid^1^	Urine	N	Y	Y	U	U	U	Y	N	N
Basil	Estragole	Plasma	N	Y	Y	U	U	N	U	Y	N
	Estragole	Urine	N	Y	Y	U	U	N	U	Y	N
	*trans*-anethole	Plasma	N	Y	Y	U	U	Y	U	Y	N
	*trans*-anethole	Urine	N	Y	Y	U	U	Y	U	Y	N
	*para-Anisaldehyde*	Plasma	N	Y	Y	U	U	N	U	Y	N
	*para-Anisaldehyde*	Urine	N	Y	Y	U	U	N	U	Y	N
*Capsicum* (chili pepper)	Capsaicin	Plasma	Y	Y	Y	U	U	U	U	U	N
*Capsicum* (CH-19 sweet non-pungent red pepper)	Capsiate	Plasma	Y	U	N	U	U	U	U	N	N
	Dihydrocapsiate		Y	U	N	U	U	U	U	N	N
	Nordihydrocapsiate		Y	U	N	U	U	U	U	N	N
	Vanillyl alcohol		N	U	N	U	U	U	U	N	N
*Capsicum* (paprika carotenoids)	β-Cryptoxanthin	Plasma and erythrocy-tes	N	Y	N	Y	U	N	U	N	N
	Cucurbitaxanthin A		Y	Y	N	Y	U	U	U	N	N
	Lutein		N	Y	N	Y	U	N	U	N	N
	Zeaxanthin		N	Y	N	Y	U	N	U	N	N
	Capsanthin		Y	Y	N	Y	U	U	U	N	N
	Capsanthone		Y	Y	N	Y	U	U	U	N	N
	Cryptocapsin		Y	Y	N	Y	U	U	U	N	N
Cinnamon	7-hydroxycoumarin	Plasma	Y	Y	Y	U	Y	U	Y	Y	N
	7-hydroxycoumarin	Urine	Y	Y	Y	U	Y	U	Y	Y	N
Fennel	*trans*-Anethole	Plasma	N	Y	Y	U	U	Y	U	Y	N
	*trans*-Anethole	Urine	N	Y	Y	U	U	Y	U	Y	N
	1′-hydroxyestragole-G	Plasma	N	Y	N	U	U	U	U	Y	N
	1′-hydroxyestragole	Urine	N	Y	N	U	U	U	Y	Y	N
	1′-hydroxyestragole-G	Urine	N	N	N	U	U	U	U	Y	N
	*p*-Allylphenol-G	Plasma	N	N	N	U	U	U	U	Y	N
	*p*-Allylphenol-G	Urine	N	N	N	U	U	U	U	Y	N
	Estragole	Plasma	N	Y	Y	U	U	N	U	Y	N
	Estragole	Urine	N	Y	Y	U	U	N	U	Y	N
	*para*-Anisaldehyde	Plasma	N	Y	Y	U	U	Y	U	Y	N
	*para*-Anisaldehyde	Urine	N	Y	Y	U	U	Y	U	Y	N
Ginger	10-Gingerol	Plasma	Y	Y	Y	N	U	U	Y	Y	N
	6-Shogaol		Y	Y	Y	N	U	U	Y	Y	N
	6-Gingerol -G		Y	Y	Y	N	U	U	Y	Y	N
	8-Gingerol-G		Y	Y	Y	N	U	U	Y	Y	N
	10-Gingerol-G		Y	Y	Y	N	U	U	Y	Y	N
	6-Shogaol-G		Y	Y	Y	N	U	U	Y	Y	N
	6-Gingerol-S		Y	Y	Y	N	U	U	Y	Y	N
	8-Gingerol-S		Y	Y	Y	N	U	U	Y	Y	N
	10-Gingerol-S		Y	Y	Y	N	U	U	Y	Y	N
	6-Shogaol-S		Y	Y	Y	N	U	U	Y	Y	N
Marjoram (*Origanum onites*)	*p*-hydroxybenzoic acid	Urine	N	Y	Y	U	U	U	Y	Y	N
	Vanillic acid		N	Y	Y	U	U	U	Y	Y	N
	*m*-hydroxyphenylacetic acid		N	Y	Y	U	U	U	Y	Y	N
	Ferulic acid		N	Y	Y	U	U	U	Y	Y	N
	3,4-dihydroxyphenylacetic acid		N	Y	Y	U	U	U	Y	Y	N
	Protocatechuic acid		N	Y	Y	U	U	U	Y	Y	N
	Syringic acid		N	Y	Y	U	U	U	Y	Y	N
	Caffeic acid		N	Y	Y	U	U	U	Y	Y	N
	*p*-coumaric acid		N	Y	Y	U	U	U	Y	Y	N
Nutmeg	O-demethyl elemicin	Urine	Y	Y	N	U	U	Y	U	N	N
	O-demethyl dihydroxy elemicin		Y	Y	N	U	U	Y	U	N	N
	Demethylenyl myristicin		Y	Y	N	U	U	Y	U	N	N
	Dihydroxy myristicin		Y	Y	N	U	U	Y	U	N	N
	Demethylenyl safrole		Y	Y	N	U	U	Y	U	N	N
Parsley	Apigenin	Plasma	N	Y	Y	U	U	U	Y	U	N
	Apigenin	Red cells	N	N	Y	U	U	U	Y	U	N
	Apigenin	Urine	N	Y	Y	U	U	U	Y	Y	N
Peppermint (capsule, oral admin.)	Menthol glucuronide	Urine	Y	Y	Y	U	U	U	Y	Y	N
Peppermint (intragastric spray)	Menthol	Plasma	Y	Y	Y	U	U	U	U	Y	N
	Menthol glucuronide	Plasma	Y	Y	Y	U	U	U	Y	Y	N
	M7	Plasma	U	Y	Y	U	U	U	Y	Y	N
	M9	Plasma	U	Y	Y	U	U	U	Y	Y	N
	M11	Plasma	U	Y	Y	U	U	U	Y	Y	N
	M29	Plasma	U	Y	Y	U	U	U	Y	Y	N
	M2–16, M18–21, M24, M29–31	Urine	U	U	Y	U	U	U	Y	Y	N
Saffron	Crocetin	Plasma	Y	Y	Y	U	U	U	Y	Y	N
Sage	1,8 cineole	Plasma	N	Y	U	U	U	U	N	Y	N
	1,8 cineole	Urine	N	Y	N	U	U	U	N	Y	N
	2-OH-1,8-cineole	Plasma	N	Y	N	U	U	U	N	Y	N
	2-OH-1,8-cineole	Urine	N	Y	N	U	U	U	N	Y	N
	3-OH-1,8-cineole	Plasma	N	N	N	U	U	U	N	Y	N
	3-OH-1,8-cineole	Urine	N	Y	N	U	U	U	N	Y	N
	7-OH-1,8-cineole	Plasma	N	N	N	U	U	U	N	Y	N
	7-OH-1,8-cineole	Urine	N	Y	N	U	U	U	N	Y	N
	9-OH-1,8-cineole	Plasma	N	Y	N	U	U	U	N	Y	N
	9-OH-1,8-cineole	Urine	N	Y	N	U	U	U	N	Y	N
Tarragon	Estragole	Plasma	N	Y	Y	U	U	N	U	Y	N
	Estragole	Urine	N	Y	Y	U	U	N	U	Y	N
	*trans*-anethole	Plasma	N	Y	Y	U	U	Y	U	Y	N
	*trans*-anethole	Urine	N	Y	Y	U	U	Y	U	Y	N
	*para-Anisaldehyde*	Plasma	N	Y	Y	U	U	Y	U	Y	N
	*para-Anisaldehyde*	Urine	N	Y	Y	U	U	Y	U	Y	N
Thyme	Thymol-S	Plasma	N	Y	Y	U	U	Y	Y	Y	N
	Thymol-S	Urine	N	Y	Y	U	U	Y	Y	Y	N
	Thymol-G	Urine	N	Y	Y	U	U	Y	Y	Y	N
	Caffeic acid-S	Urine	N	Y	N	U	U	Y	Y	Y	N
	OH-phenylpropionic acid-S	Plasma	N	Y	N	U	U	Y	Y	Y	N
	OH-phenylpropionic acid-S	Urine	N	Y	N	U	U	Y	Y	Y	N
	*p*-cymene-diol-G	Plasma	Y	Y	N	U	U	Y	Y	Y	N
	*p*-cymene-diol-G	Urine	Y	Y	N	U	U	Y	Y	Y	N
	Carvacrol	Feces	N	N	N	U	U	U	Y	U	N
	2-(4-hydroxyphenyl)acetic acid	Feces	U	N	N	U	U	U	Y	U	N
	3-(3′-4′-dihydroxyphenyl)propionic acid	Feces	U	N	N	U	U	U	Y	U	N
	OH-phenylpropionic acid	Feces	Y	N	N	U	U	U	Y	U	N
	3-phenylpropionic acid	Feces	U	N	N	U	U	U	Y	U	N
Turmeric (curcuminoids in capsule, or nanoemulsion)	Curcumin	Plasma	Y	Y	Y	U	Y	U	Y	Y	Y
	Curcumin	Serum	Y	Y	Y	U	U	U	Y	N	N
	Curcumin	Urine	Y	Y	U	U	U	U	Y	U	N
	Curcumin	Feces	Y	Y	U	U	U	U	Y	U	N
	Curcumin	Colon	Y	Y	Y	Y	Y	Y	Y	U	N
	DMC	Plasma	Y	Y	Y	U	Y	U	Y	Y	Y
	DMC	Urine	Y	Y	U	U	U	U	Y	U	N
	DMC	Colon	Y	Y	U	Y	U	U	U	U	N
	DMC-G	Plasma	Y	Y	U	Y	U	U	U	U	N
	DMC-G	Urine	Y	Y	U	Y	U	U	U	U	N
	DMC-S	Plasma	Y	Y	U	Y	U	U	U	U	N
	BDMC	Plasma	Y	Y	Y	U	Y	U	Y	Y	Y
	BDMC	Urine	Y	Y	U	U	U	U	Y	U	N
	BDMC	Colon	Y	Y	U	Y	U	U	U	U	N
	THC	Plasma	Y	Y	Y	U	Y	U	Y	Y	Y
	COG	Plasma	Y	Y	Y	U	U	U	Y	Y	Y
	COG	Urine	Y	Y	U	U	U	U	Y	U	N
	COG	Colon	Y	Y	U	Y	U	U	U	U	N
	COS	Plasma	Y	Y	Y	U	Y	U	Y	Y	Y
	COS	Urine	Y	Y	U	U	U	U	Y	U	N
	COS	Colon	Y	Y	U	Y	U	U	U	U	N
	COS	Feces	Y	Y	U	U	U	U	Y	U	N
	Hexahydrocurcumin	Liver	Y	Y	N	U	U	U	Y	U	N
	Hexahydrocurcuminol	Liver	Y	Y	N	U	U	U	Y	U	N
	Ferulic acid	Plasma	N	N	N	U	Y	U	N	N	N
	Vanillic acid	Plasma	N	N	N	U	Y	U	N	N	N
Turmeric (Theracurmin^1^)	Curcumin	Plasma	Y	Y	Y	U	U	U	U	U	N
Turmeric-fresh curcuminoids (capsule)	Curcumin	Plasma	Y	Y	Y	U	U	U	Y	U	N
	DMC	Plasma	Y	Y	N	U	U	U	U	U	N
	BDMC	Plasma	Y	Y	N	U	U	U	U	U	N

*−G*, glucuronide; *−S*, sulfate; *BMDC*, bisdemethoxycurcumin; *DMC*, demethoxycurcumin; *DMC-G*, demethoxycurcumin glucuronide; *THC* tetrahydrocurcumin; *COG* curcumin-O-glucoronide. *COS*, curcumin-O-sulfate. *M29*, menthol sulfoconjugate; hydroxyl menthol; *M7, 9, 11* glucuronide; *M3–11*, hydroxyl menthol glucuronide; *M19–21*, dihydroxyl menthol glucuronide; *M2*, aldehyde-menthol glucuronide; *M13–18* carboxylate-menthol, or aldehyde-hydroxyl menthol glucuronide; *M29–32*, sulfate conjugates; *M12*, dialdehydementhol glucuronide

^**1**^Theracurmin, commercialized nanoparticle curcumin to increase its absorption

^*^Possible answers are *Y* (yes), *N* (no), or *U* (unknown or uncertain, or not specified in the study)

*Biological/nutritional validation and applicability*:

1. Is the marker compound known as unique for the food or food group (chemical/biological plausibility)?

2. Is there a dose-response relationship at relevant intake levels of the targeted food (quantitative aspect)?

3a. Is the single-meal time-response relationship described adequately (single-dose kinetics)?

3b. Is the biomarker kinetics for repeated intakes of the food/food group adequate (e.g., cumulative aspects)?

4. Has the marker been shown to be robust after intake of complex meals (robustness)?

5. Has the marker been shown to compare well with other markers for the same food/food group (reliability)?

*Analytical validation*:

6. Is the marker chemically and biologically stable, making measurement reliable (feasibility)?

7. Are analytical variability (CV%), accuracy, sensitivity, and specificity known as adequate (analytical performance)?

8. Has the analysis been successfully reproduced in another laboratory (reproducibility)?

## Results

**Fig. 1:**
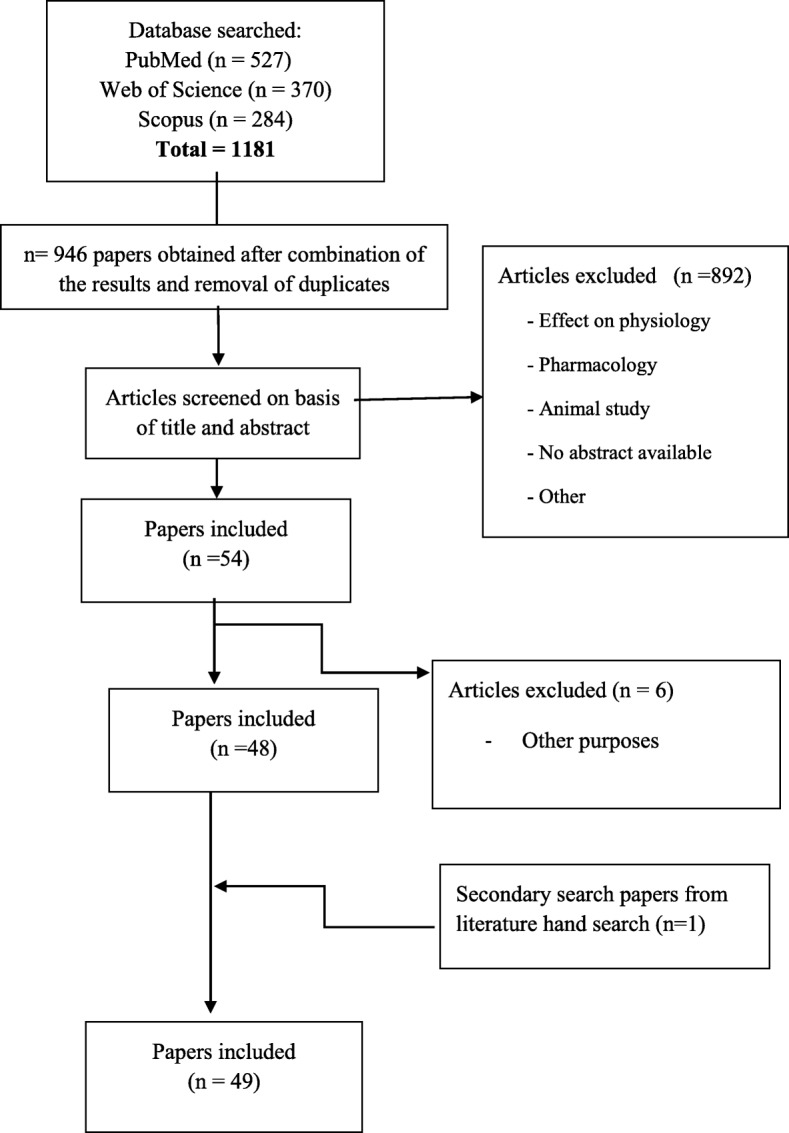
Flow diagram of the study selection

The research papers that identified, described, or evaluated potential biomarkers of intake for the set of 25 herbs and spices were further screened by one or more skilled researchers as described in Fig. 1. The initial PubMed search retrieved 527 matches, the Web of Science search generated 370 matches, and the Scopus search generated 284 matches, resulting in a total of 1181 hits. This number was reduced to 946 after the removal of duplicates. Subsequent screening of the titles and abstracts by the skilled researchers reduced the number of papers to 54. Further evaluation excluded 6 papers and a secondary search identified 1 more paper leading to a total of 49 papers that were included in this review (Fig. 1). Additional papers were identified from the reference lists in these papers and from reviews or book chapters identified through the literature search. This secondary search was used to evaluate the apparent specificity of the marker.

From this total of 49 papers, 18 BFI papers were found for turmeric and curcumin, 6 papers were found for peppermint, 4 papers were found for thyme, 3 papers for ginger, *Capsicum* and parsley, 2 papers for anise and saffron; and 1 paper each was found for cinnamon, fennel, nutmeg, oregano, marjoram, rosemary and sage, and finally one paper which examined basil, tarragon and fennel (Table 2). No potential BFI papers were found for black pepper, caraway, clove, cumin, dill, fenugreek, lemongrass, and spearmint.

**Fig. 2:**
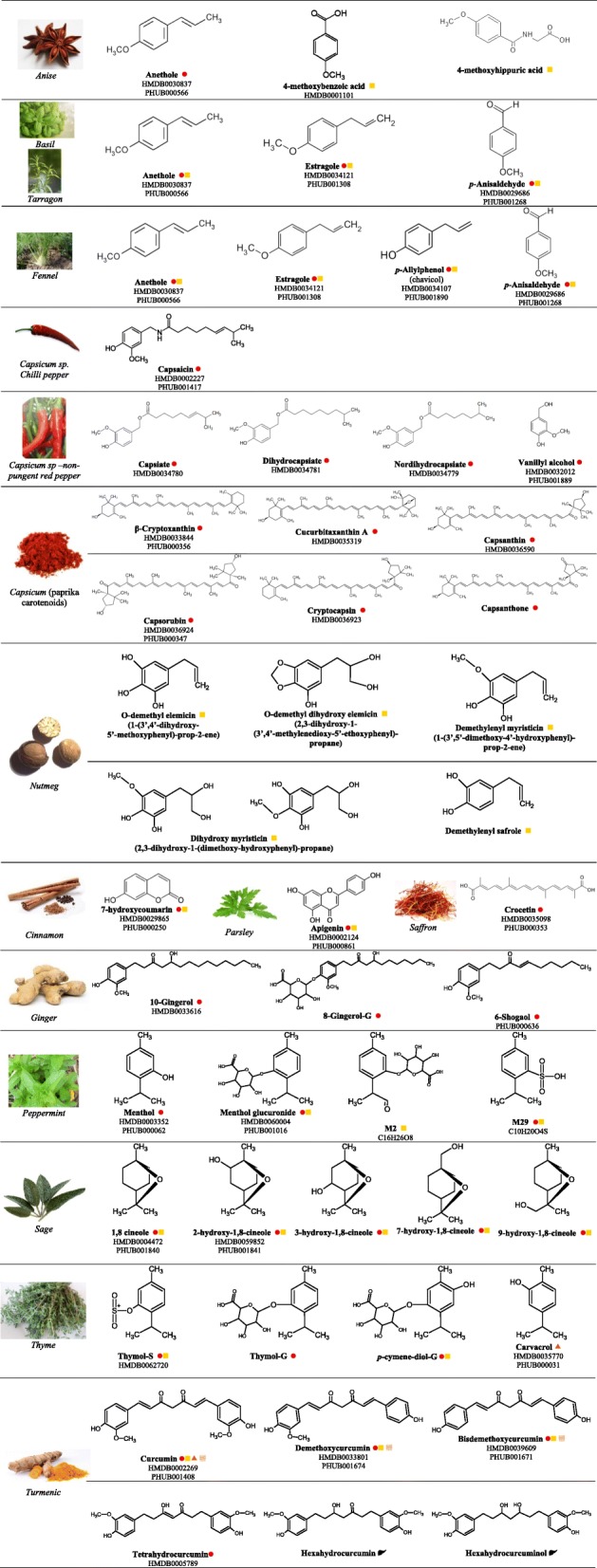
Examples of the most representative metabolites for each herb/spice identified in the reviewed studies. HMDB ID; phytohub code (PHUB); −G, glucuronide; −S, sulfate; aldehyde-menthol glucuronide (M2); menthol sulfate conjugates (M29);  in the blood;  in urine;  in feces;  in the intestine;  in the liver

A systematic validation of the candidate intake biomarkers is assessed and presented in Table 3. It should be noted that the search criteria were primarily targeted for BFIs in humans, but, in some cases, a small number of papers in which the BFI study was performed in animals were also investigated. These papers were used to provide supportive information to the studies performed in humans; however, the biomarkers observed only in animal studies were not considered eligible for further BFI validation. Figure 2 summarizes many of the findings of this review. It includes the most representative or strongly validated metabolites identified for each herb and spice, the molecule formula, the HMDB ID, and/or Phytohub code (if exists) and the biofluid or tissue in which each BFI has been found.

### Anise

Anise is a seed spice derived from a flowering plant belonging to the family *Apiaceae*, which is native to the Eastern Mediterranean region and Southwest Asia. The distinctive licorice flavor and aroma from anise comes from anethole. Anethole is a phenylpropene derivative found in anise (*Pimpinella anisum*) and fennel (*Foeniculum vulgare*). Anethole occurs naturally in high concentrations in volatile oils such as anise oil (80–90%), star anise oil (over 90%), and fennel oil (80%) [34]. Anethole exists in both a *cis* and a *trans* isomer with the *trans* isomer being more abundant. It is the main component of the anise essential oil (80–90%), with minor components including para-anisaldehyde, estragole, and pseudoisoeugenyl-2-methylbutyrates, among others [35]. In Mediterranean countries, the popularity of alcoholic and non-alcoholic anise-flavored beverages has led to a much greater consumption of *trans-*anethole [36]. Anethole is also used in medicines as an expectorant, an antitussive and an antispasmodic for treating gastrointestinal tract illnesses. As a result, anise is found in a number of pharmaceutical products.

Just two papers have reported potential anise intake biomarkers. The most complete study was conducted in 1988 [37], where Caldwell and co-workers performed an acute human study administrating *trans*-anethole (using a synthesized radio-labeled ^14^C compound). The major routes of elimination of ^14^C were in the urine (54–69% of the administered dose) and as exhaled ^14^CO_2_ (13–17%). The principal metabolite (> 90% of urinary ^14^C) was 4-methoxyhippuric acid (also known as anisic acid), accompanied by much smaller amounts of 4-methoxybenzoic acid (or also known as *p*-anisic acid, an oxidation product of anethole) and up to three other unknown compounds. As the authors stated, metabolism of anethole in man was unaffected by changes in dose size, and is dominated by the ɷ-oxidation pathway ultimately leading to 4-methoxyhippuric acid, and by oxidative O-demethylation, leading to the exhalation of ^14^CO_2_ [36].

The only other research article to look into anise BFIs was an observational study that measured the content of anethole in the blood after the intake of alcoholic anise-based beverages [37]. This study rigorously monitored a single individual, wherein the subject consumed the alcoholic drink ouzo over three different days under controlled conditions. In addition to this controlled single-participant study, the authors also looked at the blood collected from 50 motor vehicle drivers who claimed to have consumed drinks containing anethole (ouzo, raki and the German aniseed liqueur “Küstennebel”). The anethole concentrations detected for the tested volunteer showed rapid resorption of anethole as well as rapid elimination. Anethole concentrations above the detection level of 3.6 ng/ml serum were detected in the selected volunteer for 3 h after ceasing consumption of 120 ml of Helenas ouzo and for 3 h after ceasing consumption of 200 ml of regular ouzo, and for 7 h after ceasing consumption of 360 ml of regular ouzo. For the 50 motor vehicle drivers, 10 out of 50 serum samples had anethole concentrations of between 5.4 and 17.6 ng/ml. Of these, eight corresponded to confirmed cases of ouzo consumption, one of raki consumption and one of German aniseed liqueur “Kustennebel” consumption. The authors concluded that anethole can be reliably detected in blood/serum samples after consumption of spirits containing anethole. In no case was a positive result for anethole found where 40 ml or less of spirits containing anethole had been consumed or where the time difference between the cessation of drinking and the taking of the blood sample was greater than 4 h.

Based on these two studies, we can conclude that anethole seems to be a robust and reliable blood BFI for anise consumption as assessed by observational studies involving the consumption of anise-based drinks. While a single, high-quality marker for specific food intake is ideal, the addition of other (unrelated) biomarkers to create a multi-component biomarker panel can substantially improve a biomarker’s sensitivity and specificity [33]. In this regard, two compounds, 4-methoxyhippuric acid and 4-methoxybenzoic acid have been specifically detected in urine after direct anethole intake. However, these two compounds are actually metabolites of anethole and so are unlikely to add to anethole’s sensitivity/specificity. We also believe that further studies are needed to confirm that these two compounds are seen with actual anise-based food intake. It is also worth noting that anethole is found in fennel, basil, and tarragon [38], and it is widely present in pharmaceutical products and as a flavoring additive. Therefore, anethole and its metabolites may not be sufficiently specific BFIs for anise intake.

### Capsicum sp.

#### Chili pepper

The chili pepper is a fruit spice derived from plants from the genus *Capsicum*, originated in Mexico and brought to Asia by Portuguese navigators during the sixteenth century. The five domesticated species of pepper are *Capsicum annuum*, *C. frutescens, C. chinense*, *C. pubescens*, and *C. baccatum.* Chili peppers have a taste that is pungent, hot, and somewhat sweet (depending on the variety and type). Mild or sweet peppers contain similar constituents as *Capsicum* but with little or no pungent components. Chili peppers are used as food colorants, flavoring agents, as predator repellants, and a source of pain relief. The compounds responsible for the “hot” flavor of chili peppers are called capsaicinoids, with capsaicin being the best known. Capsaicin occurs naturally in plants of the *Solanaceae* family. It is commonly used in both food and medicine, but its strong pungency limits the quantity that can be employed. *Capsicum* contains up to 1.5% (by weight) of pungent compounds, commonly composed of capsaicin, dihydrocapsaicin, and others. Other constituents present in chili peppers are carotenoids, vitamins A, C, and small amounts of volatile oils with more than 125 known components. Another class of capsaicin-like compounds found in chili peppers and non-pungent chili peppers are the capsinoids. Capsinoids have an estimated “hot taste threshold” that is about 1/1000 that of capsaicin making it possible to use capsinoids in food applications without the intense heat effect found in capsaicins [39]. Many positive health benefits have been ascribed to both capsaicin and capsinoids, including anticancer, anti-inflammatory, and analgesic effects [40].

We found two published studies that explored possible BFIs associated with chili pepper consumption. In a cross-over study with 12 volunteers [41], capsaicin was detected in plasma by HPLC analysis after the administration of 5 g of capsaicin derived from chili pepper. This pharmacokinetic study showed that capsaicin was rapidly absorbed (being detected at 10 min) after ingestion and also rapidly metabolized (not detected in blood after 90 min). Another study, conducted by Bernard et al. [42], analyzed the metabolites present in plasma by LC-MS/MS after the administration of a capsule of a variety of non-pungent (sweet) pepper (CH-19) extract. The compounds identified were the capsinoids capsiate, dihydrocapsiate and nordihydrocapsiate, and a capsinoid metabolite, vanillyl alcohol. However, these compounds were below the limit of quantification, so the authors could not perform proper kinetic studies.

Based on these data, capsaicin, the main compound responsible for the hot taste of chili peppers, can be considered a specific BFI for chili peppers. However, we believe more data are required and further studies should be performed to confirm the utility of capsaicin as a BFI. With regard to the non-pungent compounds of the “sweet” varieties of *Capsicum*, (i.e., capsinoids) additional dose-response studies are needed to consider them as plausible BFIs since the concentrations measured in the single reported study were too low to perform kinetic analyses.

#### Paprika

Paprika is a ground spice made from the red, air-dried fruits of the larger and sweeter varieties of the plant *Capsicum annuum*, which is also called bell pepper or sweet pepper. Paprika can also be modified with the addition of more pungent chili peppers and cayenne pepper. Originating in central Mexico, paprika was brought to Spain in the sixteenth century. Paprika spices can range from mild to hot, depending on the variety of the source plant. The flavor also varies from country to country—but almost all plants grown produce the sweet variety. The red, orange, or yellow color of paprika is due to its content of carotenoids. The intense color of paprika makes it an ideal and natural food colorant for many dishes.

Only one study was found that looked into the identification of biomarkers of food intake for paprika. Nishino and co-workers [42] measured paprika carotenoids in the plasma and erythrocytes of five volunteers who were supplemented with paprika for 4 weeks. The results showed that several, non-unique carotenoids such as lutein, zeaxanthin, *β*-cryptoxanthin (also found in vegetables such as carrots and tomatoes) could be detected in the paprika-supplemented volunteers. However, the authors also detected other carotenoids specific for paprika, such as cryptocapsin, capsanthin, capsorubin (just found in paprika and lily pollen), cucurbitaxanthin A (found just in paprika and pumpkins), and finally capsanthone (a possible oxidative product of capsanthin). Paprika carotenoids, particularly capsanthin and capsorubin, have been reported to have a strong antioxidant activity [43, 44]. Based on these results, cucurbitaxanthin A, capsanthin, capsanthone, and cryptocapsin could be potential paprika-specific carotenoid biomarkers. However, further analyses using untargeted MS-based approaches should be conducted to evaluate other possible biomarkers of paprika intake.

#### Cinnamon

Cinnamon is a bark spice obtained from the inner bark of several tree species from the genus *Cinnamomum*. Only a few *Cinnamomum* species are grown commercially (largely from Asia) for spice. Cinnamon is native to India, Sri Lanka, Bangladesh, and Myanmar, and it was imported to Egypt as early as 4000 years ago [45]. In addition to its common culinary use as a condiment and flavoring material, cinnamon is widely known for its anti-diabetic and glucose lowering effects [46]. The flavor of cinnamon is due to an aromatic essential oil that is largely composed of cinnamaldehyde (up to 90%); however, there are at least 80 other compounds known to be in cinnamon oil, including cinnamyl alcohol, cinnamyl acetate, eugenol, and various coumarins that contribute to its overall flavor and aroma [47].

Only one study has been performed in humans to identify BFIs of cinnamon intake [48]. While cinnamaldehyde might have been expected to be a useful BFI, it is quickly metabolized to cinnamic acid [49], making it unusable as a cinnamon biomarker. On the other hand, coumarin was deemed to be a potentially useful BFI for cinnamon. Because coumarin has a very strong first-pass effect in the liver, with only a small percentage reaching systemic circulation, the authors chose its main metabolite, 7- hydroxycoumarin as a measure of relative bioavailability. In the study by Abraham et al. [40], 7-hydroxycoumarin was assessed as a biomarker of cinnamon consumption in both urine and plasma via HPLC-MS/MS analysis. The conversion of coumarin to 7-hydroxycoumarin is catalyzed by cytochrome P450 2A6 (CYP2A6) [50]. 7-hydroxycoumarin and its phase II metabolite, 7-hydroxycoumarin glucuronide, are rapidly excreted via the kidneys [51]. Therefore, the total amount of 7-hydroxycoumarin (free and bound as a glucuronide) in urine could serve as an indirect measure of the extent of cinnamon consumption.

Coumarin possesses a pleasant spicy odor of fresh hay or vanilla [48]. The occurrence of coumarin has been reported in a number of bedding plants such as *Anthoxanthum odoratum* (sweet vernal grass), *Asperula odorata* (sweet woodruff), *Dipterix odorata* (tonka bean), *Eupatorium triplinerve* (white snakeroot), *Hierochloe odorata* (holy grass), *Melilotus coerulea* (sweet trefoil), *M. officinalis* (common melilot), *Melittis melissophyllum* (bastard balm), *Primula elatior* (oxlip), and *Trilisa odoratissima* (deer tongue). However, none of these plants are usually used as edible foods; thus, the main source of coumarin in the diet is cinnamon [52]. Coumarin, which is frequently used in perfumes, is also a well-known hepatotoxin (based on animal studies). Interestingly, different species of cinnamon have different levels of coumarin. For example, *C. cassia* cinnamon contains up to 1% coumarin, whereas the more expensive and less frequently used true cinnamon (*Cinnamomum verum*) contains only trace levels (0.004%) [53, 54]. Today, many commercially available food products are spiced with the cheaper *C. cassia* cinnamon and consequently contain high levels of coumarin. It is notable that German Christmas cookies (which contain considerable amounts of *C. cassia* cinnamon) have a coumarin content that often exceeds the maximum tolerable dose intake (TDI 0.1 mg/kg body weight).

Based on the available data and based on the fact that the food matrix effect has been well tested, 7-hydroxycoumarin appears to be a plausible specific and robust biomarker of cinnamon intake. Additional cumulative/kinetic aspects of this biomarker need to be performed and most likely an inter-laboratory validation needs to be completed to fully validate this compound as a cinnamon BFI. However, the use of this metabolite as a BFI for cinnamon is confounded by the fact that it depends on the species of cinnamon being used. We believe that other cinnamon compounds (such as cinnamaldehyde and cinnamic acid or the essential oils as cinnamyl alcohol, cinnamyl acetate and eugenol)) should also be explored as potential biomarkers as, so far, the only reported cinnamon BFI study was limited to measuring coumarin and its derivatives.

#### Fennel, basil, and tarragon

While fennel, basil, and tarragon are very distinct herbs, coming from very different plant species, they share a number of common chemicals and consequently they tend to be grouped together in food intake studies. This is why we have chosen to group these three herbs under a single topic heading.

Fennel is a seed (and bulb) spice, as well as a leaf herb, that is derived from *Foeniculum vulgare*. This is a small flowering plant that was originally indigenous to the shores of the Mediterranean, but which has since become widely naturalized in many parts of the world. Fennel is a highly aromatic and flavorful herb/spice and is one of the primary ingredients of absinthe. The distinctive licorice flavor and aroma from fennel comes from anethole. Other compounds known to be in fennel include estragole, fenchone, 1,8-cineole (eucalyptol), and *p*-allylphenol. In addition to its use in culinary applications, fennel has long been used as a medicinal herb to treat gastrointestinal illnesses and upper respiratory tract infections as well as to increase milk production in breastfeeding mothers through the consumption of fennel tea.

Basil (*Ocimum basilicum*) is a culinary herb belonging to the botanical family *Lamiaceae*. It is a culinary herb that is prominently featured in Italian cuisine as well as many Southeast Asian cuisines. Depending on the species and cultivar, the leaves may taste somewhat like anise, with a strong, pungent, often sweet smell. Thai basil is also a condiment in the Vietnamese noodle soup. Basil has been used traditionally as a medicinal herb in the treatment of headaches, coughs, diarrhea, constipation, warts, worms, and kidney disorders [55]. It is also a source of aroma compounds and essential oils containing biologically active constituents that possess antimicrobial and antifungal properties [56, 57]. Linalool is the main constituent of the essential oil of *O. basilicum* (28.6–60.6%), followed by estragole, methyl cinnamate, epi-α-cadinol, α-bergamotene, γ-cadinene (3.3–5.4%), germacrene D (1.1–3.3%), and camphor (1.1–3.1%). Other compounds such as myrcene, pinene, terpineol, 1,8-cineole, eugenol, and methyleugenol have been identified in basil leaves [56, 58, 59].

Tarragon (*Artemisia dracunculus*), also known as estragon, is a perennial herb belonging to the *Asteraceae* (daisy) family. It is widespread across much of Eurasia and North America, and is cultivated for culinary and medicinal purposes. Two well-described “cultivars” (Russian and French) are widely used. “Dracunculus” which in Latin meaning “little dragon” is believed to describe its coiled, serpentine root, and/or the shape of the leaves, which is reminiscent of a dragon’s tongue [60]. In vitro pharmacological studies indicate that tarragon has antibacterial, antifungal, and antiplatelet activity [61]. In vivo pharmacological studies have shown that tarragon has anti-inflammatory, hepatoprotective, antihyperglycemic, and antioxidant activity [61]. The major components of Russian tarragon are reported to be terpinen-4-ol, sabinene, and elemicin. Methyleugenol and estragole are usually present in tarragon oils at about 10 and 3%, respectively. However, estragole is one of the predominant compounds in the essential oil of French tarragon, constituting up to 82% [61]. *Trans*-anethole (21.1%), α-trans-ocimene (20.6%), limonene (12.4%), α-pinene (5.1%), and allo-ocimene (4.8%) are the other main components of tarragon [62].

Two studies have explored or assessed potential biomarkers of fennel (alone) or fennel, tarragon, and basil intake in humans. The first one, by Zeller et al. [63], studied the metabolism of estragole in humans consuming fennel tea. The metabolites identified in the urine of subjects were estragole, 1′-hydroxyestrogle, *trans*-anethole (also reported in anise [36, 37]), and *p*-allylphenol (also found in betel leaf oils and in oil of bay). However, the authors were unable to report concentrations for these compounds or to correlate them with fennel dosage. In terms of the specificity of these compounds, estragole, in addition to being found in fennel, is a known component of several herbs such as tarragon, basil, and anise. Estragole, which is structurally similar to safrole, is rapidly metabolized to 1′-hydroxyestragole and is quickly excreted as its glucuronic acid conjugate.

In the second study by Barfi et al. [38], the authors developed and validated a multi-step method to extract *trans*-anethole, estragole, and *para*-anisaldehyde (three major components of fennel, basil and tarragon) from biofluids and then applied this extraction technique to real human plasma and urine samples. All three compounds were found in plasma and urine after the consumption of either 15 ml of fennel extract, 15 ml of tarragon extract or 15 ml of brewed basil.

While studies of fennel, basil, and tarragon phytochemicals and essential oils have identified several potentially unique compounds for each of these herbs, the same cannot be said of the BIFs that have been, so far, identified. To date, all of the food intake compounds identified for fennel, basil, and tarragon consumption [38, 63] are not sufficiently specific to identify one from the other or any of them from other widely consumed herbs. This is because all of the reported compounds found in human biofluids, so far, are also found in other herbs and spices (such as anise). Therefore, we conclude that no specific BFI for fennel, basil, or tarragon intake has, to date, been discovered or described in the literature.

#### Ginger

Ginger (*Zingiber officinale*) is a root or rhizome-based spice derived from the ginger plant, a member of the turmeric family (both are from *Zingiberaceae*). Ginger is believed to have originated in India and is widely used as a culinary additive as a hot, fragrant spice as well as a popular medicine. In addition to ginger’s well-known use as a treatment for nausea, many components in ginger have been found to have anti-inflammatory, antibacterial, antipyretic, antilipidemic, antitumorigenic, and antiangiogenic effects [64–66]. Ginger’s flavor and aroma come from its volatile oils (∼1 to 3% of the weight of fresh ginger) and non-volatile pungent oleoresins. A variety of active components have been identified in the oleoresins of ginger including zingerone, gingerols (6-, 8-, and 10-gingerols), and shogaols (6-, 8-, and 10-shogaols) [67]. Gingerols (especially 6-gingerol) are the major pungent components in the fresh ginger rhizome. In dried ginger, the quantity of shogaols are significantly increased as evidenced by the reduction of the ratio of 6-gingerol to 6-shogaol from 10:1 in fresh ginger to 1:1 in dried ginger [68]. In particular, zingerone is produced from gingerols during drying, having lower pungency and a spicy-sweet aroma.

A total of three studies have been reported on potential BFIs for ginger or ginger extracts. The earliest study by Zick et al. [69] found that 6, 8-, and 10-gingerols and 6-shogaol are absorbed after oral ginger extract dosing and can be detected as glucuronide and sulfate conjugates [69]. Yu and co-workers [55] detected free 10-gingerol and 6-shogaol in the human plasma, whereas the majority of the 6-, 8-, and 10-gingerols and 6-shogaol existed as glucuronide and sulfate metabolites after oral dosing of 2 g ginger extracts [70]. No free 6-gingerol was detected in plasma despite it being the most abundant component of ginger extracts (2.64%). In comparison, although 6-shogaol makes up 2.25% and 10-gingerol only accounts for 1.22% of most ginger extracts, 6-shogaol and 10-gingerol were readily detected in human plasma. Pharmacokinetic studies showed very short half-lives for these four analytes and their metabolites (1–3 h in human plasma). Due to their short half-lives, no accumulation was observed for 6-, 8-, and 10-gingerols and 6-shogaol (and their metabolites) in either plasma or colon tissues even after multiple daily dosing. A third biomarker intake study was focused on the metabolism of shogaol [60]. The results of this study (Table 2) show that it was possible to detect all the major thiol-conjugated metabolites of shogaol in human urine using LC-MS/MS [71]. The authors suggested the mercapturic acid pathway as a major metabolic route for shogaols in humans.

Based on the available data, 6-, 8-, and 10-gingerol glucuronides and sulfates along with 6-shogaols appear to be plausible, specific, and robust biomarkers of ginger intake. More studies are needed to confirm that they are also seen with actual ginger-based food intake. Additional cumulative/kinetic aspects of these biomarkers need further evaluation and most likely an inter-laboratory validation is required to make these compounds fully validated BFIs for ginger consumption.

#### Nutmeg

Nutmeg is a fragrant flavoring spice coming from the seed of *Myristica fragrans (*belonging to the *Myristicaceae* family*),* an evergreen tree indigenous to the Banda Islands in the Moluccas (or Spice Islands) of Indonesia. Until the mid-nineteenth century, the small island group of the Banda Islands, was the only location of the production of nutmeg and mace in the world. This made nutmeg a particularly prized and costly spice in European medieval cuisine. The nutmeg essential oil is obtained by steam distillation of ground nutmeg, and it is used widely in the perfumery and pharmaceutical industries. This volatile fraction typically contains sabinene (21.38%), 4-terpineol (13.92%), and myristicin (13.57%), as well as portions of safrole, elimicin, terpineol, α-pinene d-camphene, limonene, linalool, and isoeugeunol [72]. Psychotropic effects have been described after ingestion of large doses of nutmeg, which are attributable to metabolic formation of amphetamine derivatives from the main nutmeg ingredients elemicin, myristicin, and safrole.

Only one study was found for the evaluation of nutmeg ingestion. Beyer and co-workers [73] evaluated nutmeg administration in animals and then performed an observational toxicological study to identify the metabolites in the urine of a human subject after the individual ingested the powder derived from 5 nutmeg seeds. In the human urine sample, the following metabolites were identified by GC-MS: O-demethyl elemicin, O-demethyl dihydroxy elemicin, demethylenyl myristicin, dihydroxy myristicin, and demethylenyl safrole. Neither amphetamine derivatives nor the main nutmeg ingredients could be detected in the rat urine nor in human urine samples [73].

Myristicin is a natural organic compound not only present in nutmeg oil, but also, to a lesser extent, in members of the *Umbelliferae* family such as carrots, parsley, celery, dill, parsnip, and black pepper [74]. The measured amount of myristicin in nutmeg and mace is very high—13,000 mg/kg (nutmeg) and 27,000 mg/kg (mace). On the other hand, it is much less in dill and parsley (1200 mg/kg and 727 mg/kg, respectively), and very low in celery (0.33 mg/kg), carrots (0.16 mg/kg), and parsnip (0.002 mg/kg) [74]. Zheng et al. [75] found that in an in vivo animal study conducted on mice, myristicin had the ability to increase the activity of the detoxifying system (potential cancer chemoprevention). This finding was replicated in another study [76]. Elemicin has been identified as an essential oil composition of carrots [77], parsley, elemi oil, banana, anise, and oregano [78]; however, the major route of elemicin intake appears to be nutmeg [79]. Lastly, safrole is a major chemical constituent (85%) of the aromatic oil of sassafras root bark (*Sassaras albidum*). It is also a minor component or trace constituent in mace, nutmeg, cinnamon, black pepper, cocoa, anise, and a number of other spices [80].

Because nutmeg is the primary known dietary source of these compounds [81], many of the metabolites of myristicin, elemicin, and safrole either alone or in combination (as a multi-marker panel) could be considered as good candidate BFIs of nutmeg intake. However, since the only study that exists on nutmeg intake is an observational study, additional controlled kinetic studies should be conducted, and further analytical performance validation should be done before these BFIs can be fully validated.

#### Oregano, marjoram, rosemary, and thyme

Oregano, marjoram, rosemary, and thyme are culinary herbs derived from members of the *Lamiacea*e plant family, which also includes basil, mint, sage, lavender, and others. Due to their phylogenetic proximity and the similarity of the compounds identified in studies of these herbs, we decided to present the results and discuss them together.

Oregano (*Origanum vulgare*) is a native herb to temperate western and southwestern Eurasia and the Mediterranean region. It has an aromatic, warm, and slightly bitter taste. Among the chemical compounds contributing to the flavor of oregano are carvacrol, thymol, limonene, pinene, ocimene, and caryophyllene [82]. Oregano also contains polyphenols, including caffeic, *p*-coumaric, and rosmarinic acid, which confer antioxidant activity and prevents lipid peroxidation [4]. It is widely used in Mediterranean cuisine, the Philippines, and Latin America, especially in Argentina. A related herb from *Origanum oonites*, which is better known as marjoram, is a plant species found in Sicily, Greece, and Turkey. Marjoram has similar flavors as oregano.

Rosemary (*Rosmarinus officinalis*) is native to the Mediterranean and Asia. The leaves are used as a flavoring agent in a variety of foods in traditional Mediterranean cuisine. They have a bitter, astringent taste, and a very characteristic aroma. Rosemary contains a number of phytochemicals, including rosmarinic acid, camphor, caffeic acid, ursolic acid, betulinic acid, carnosic acid, and carnosol [83]. Major essential oils present in rosemary oil are borneol (26.5%), α-terpinene (15.6%), and α-pinene (12.7%) [84].

Thyme (*Thymus vulgaris*) is also a member of the *Lamiaceae* family, and it has been used in foods mainly for flavor, aroma, and food preservation. Thyme has also been used in folk medicine since the times of the ancient Egyptians, Greeks, and Romans. The leafy parts of thyme are often added to meat, fish, and food products and also used as herbal medicinal products. The essential oils of common thyme contain 20–58% thymol and *p-*cymene (15–28%) as the most prevalent compounds, followed by linalool (0.7–6.5%), γ-terpinene (4–10%), carvacrol (1–4%), myrcene (1–3%), 1,8-cineole (0.8%), and borneol (0.7–1.7%) [85–87]. Thymol is the compound that provides the distinct flavor of thyme. It is also found in oregano and is used as one of many additives in cigarettes.

Oregano, rosemary, thyme, along with sage and mint are known to share several polyphenols and essential oils. Shared polyphenols include caffeic acid, chlorogenic acid, ferulic acid, *p*-coumaric acid, *p*-hydroxybenzoic acid, protocatechuic acid, and rosmarinic acid [83]. Some of the essential oils that are common to many herbs in the *Laminaceae* family are thymol (thyme, oregano, marjoram), carvacrol (thyme, oregano, marjoram), carnosic acid (rosemary and sage), carnosol (rosemary and sage), and rosmanol (rosemary and sage) [4].

Oregano, thyme, and rosemary are well known for their beneficial health properties. For example, carnosic acid and some of the diterpenes abundant in rosemary and sage appear to exert anti-obesity effects (including body weight and lipid-lowering effects) [88]. Likewise, thymol and carvacrol (oregano, thyme), carnosic acid, carnosol, rosmanol, (rosemary, sage), and epirosmanol (rosemary) have been shown to prevent lipid peroxidation and to have anti-inflammatory activity [4, 89–94]. Rosmarinic acid (found in oregano, sage, basil, rosemary, thyme and mint) exhibits anti-inflammatory effects while ferulic acid, caffeic acid, and *p*-coumaric acid inhibit LDL peroxidation [4]. Several compounds found in herbs from the *Laminaceae* family also exhibit antimicrobial activity, such as thymol, carvacrol, carnosol, rosmanol, and caffeic acid [7].

With regard to BFIs for these four herbs (thyme, marjoram, oregano, rosemary), a total of four studies were found that evaluated thyme intake, one study was found for marjoram intake, and one for oregano (evaluated in mice) and one for rosemary (evaluated in rats). In terms of oregano BFI intake, carvacrol, one of the principal components of oregano, was detected by LC-MS/MS in both murine plasma and brain after oregano extract administration [95]. Carvacrol was also found after ingestion of thyme, in a study that involved two different experimental models: (1) in vitro fermentation and (2) human intervention (in feces) [96]. These authors found that the in vitro fermentation showed limited degradation of thymol and carvacrol while the human intervention study, which used thyme phenol-enriched olive oil, increased the levels of phenylpropionic and hydroxyphenylpropionic acids in human feces, confirming in vivo microbial degradation of rosmarinic acid and eriodictyol*.* Based on these data, carvacrol can be considered a specific biomarker of thyme and oregano intake.

A human study looking at BFIs for marjoram measured urinary metabolites of 6 healthy volunteers, each of whom took a single dose of marjoram extract. In this study, the urinary metabolites identified by an HPLC-coulometric electrode array detector (CEAD) [97] were mainly polyphenol compounds such as protocatechuic acid, *p*-hydroxybenzoic, caffeic, ferulic, syringic, vanillic, *p*-coumaric, 3,4-dihydroxyphenylacetic, *m*-hydroxyphenylacetic acids. These polyphenols are also found in many other plant foods and beverages such as tea, wine, coffee, cereals, cocoa, and in general in vegetables and fruits (http://phenol-explorer.eu).

In the BFI evaluation studies of thyme, thymol [96, 98], and its derived metabolites (thymol sulfate, thymol glucuronide) [99], along with a number of polyphenols and polyphenolic metabolites including hydroxyphenyl propionic acid sulfate, coumaric acid sulfate, caffeic acid sulfate, ferulic acid sulfate, hydroxybenzoic acid dihydrophenylpropionic acid sulfate were identified in both rats [98] and humans [99]. Rosmarinic acid was also detected in plasma after thyme administration in a controlled randomized trial performed on Wistar rats [98]. Rosmarinic acid is found in both thyme and rosemary and can be considered as a biomarker of intake for both spices. Romo-Vaquero and collaborators [100] evaluated the gut, liver, plasma, and small intestine of 24 Zucker rats after both acute and subchronic administration of rosemary extract. Although the main bioactive compound of rosemary is carnosic acid [101], other derived metabolites (shown in Table 2) were also detected in this Zucker rat study. Because the two studies we found for rosemary and oregano administration were performed on animals, the compounds identified are simply being reported here for completeness. The data cannot be used to infer human BFIs for these two herbs. More studies in humans for rosemary and oregano are needed to better understand the metabolites and establish a set of specific BFI for humans.

Based on the data described here, we can conclude that several polyphenols and essential oils are shared between oregano, marjoram, rosemary and thyme. Most of these polyphenols are also common to other fruits and vegetables, so they cannot be considered specific biomarkers for these herbs. While rosmarinic acid is a very specific polyphenol for this family (*Laminaceae*), no rosamarinic acid could be detected in humans after consumption due to its rapid metabolism. With regard to essential oils, thymol and thymol sulfate in plasma and urine and thymol glucuronides in urine seem to be plausible biomarkers of thyme intake. However, these compounds are also present in oregano. Given the rather sparse number of studies done in humans for these four herbs, it is clear that additional human intervention studies are needed to evaluate if several compelling candidate biomarkers (i.e., carvacrol, carnosic acid, carnosol, rosmanol and derivatives) seen in animals can also be confirmed in human studies.

#### Parsley

Parsley (*Petroselinum crispum*) is an herb belonging to the *Apiaceae* family. It is native to the central Mediterranean region. Fresh parsley has a clean, green aroma with a versatile fresh taste that is slightly peppery with an aftertaste of green apple. Parsley is a source of several flavonoids, especially luteolin and apigenin [102, 103]. Apigenin is associated with anti-inflammatory activities as it appears to downregulate or inhibit cyclo-oxygenoase-2 (COX-2) [104]. Apigenin has also been identified as a potential cancer chemopreventive agent [105]. The major essential oil found in parsley leaves is 1,3,8-*p*-menthatriene, but other components are also present in lesser amounts including myristicin and limonene, among others [106, 107].

Two research articles were found that focused specifically on measuring BFIs of parsley [108, 109]. The first article focused on the development of a method to analyze apigenin and the apigenin metabolite acacetin (4′-methoxyapigenin) in human samples using an HPLC–UV method [108] while the second article focused on an intervention study [109] that measured these two compounds after the consumption of parsley. In this randomized crossover trial, parsley consumption together with a low flavone diet revealed a strong correlation with urinary apigenin excretion [108]. In addition to these two BFI parsley studies, several other studies have also assessed apigenin intake. One study [102], which looked at the ingestion of apigenin-rich foods like parsley, found that apigenin was elevated in urine, plasma, and red blood cells (RBCs). Although apigenin could be identified in plasma and RBCs, it was barely detectable (i.e., close to the limit of detection). On the other hand, apigenin in urine was easily identified in all individuals [102]. In another study that used a randomized crossover design for the administration of parsley, apigenin could not be detected in plasma because it was below the lower the limit of detection for the type of assay and instrument used [110].

Apigenin is a flavone not only found in parsley, but also in other vegetables from the same family (*Apiacea*), such as celery, and in lesser amounts in parsnip, carrots, and fennel. So while parsley certainly has a high content of apigenin, due to the poor specificity of this polyphenolic compound, we conclude that apigenin would not be a suitable BFI of parsley intake. Therefore, we conclude that there are no useful BFIs of parsley intake that have yet been discovered or reported in the literature.

#### Peppermint

Peppermint and spearmint are herbs that belong to the *Laminacea* family. Spearmint (*Mentha spicata*) is believed to be the oldest of the mints. It is a species of mint native to much of Europe and Asia (the Middle East, the Himalayas, China) that is now found in many parts of Northern and Western Africa, North America, and South America. On the other hand, peppermint (*Mentha × piperita*) is a hybrid mint herb. In particular, it is a cross between watermint and spearmint, and is indigenous to Europe and the Middle East. Both spearmint and peppermint have a fresh, minty, weedy, aroma. The taste is spicy, minty cool, sweet, and slightly pungent [111]. The active constituents of spearmint include spearmint oil, various flavonoids (diosmin, diosmetin), phenolic acids, and lignans. The most abundant compound in spearmint oil is carvone, which gives spearmint its distinctive smell. Spearmint oil also contains significant amounts of limonene, dihydrocarvone, and 1,8-cineol [112]. Unlike peppermint oil, spearmint oil contains minimal amounts of menthol and menthone. Conversely, peppermint has a high menthol content (40.7%), along with menthone (23.4%), and other essential oils such as menthyl acetate (4.2%), 1,8-cineole (5.3%), limonene (2.6%), menthofuran (3.7%), and β-caryophyllene (1.7%) [113, 114]. Peppermint leaves are often used alone or with other herbs in herbal teas (tisanes, infusions), ice cream, confectionery, chewing gum, toothpastes, and shampoos. Menthol activates cold-sensitive receptors in the skin and mucosal tissues, and is the primary source of the cooling sensation that follows the topical application of peppermint oil [115]. Peppermint also contains terpenoids and flavonoids such as eriocitrin, hesperidin, and kaempferol 7-O-rutinoside.

Interestingly, six research papers were found that studied the administration of peppermint (oil), but no studies were found that assessed spearmint administration. All six peppermint studies evaluated menthol and its glucuronide conjugate after administration of peppermint oil using a targeted approach. The main goal of these studies was to find a way to delay the absorption and increase the efficacy in treating spastic colon and irritable bowel syndrome. Menthol is fat-soluble and therefore is rapidly absorbed from the proximal small intestine when taken orally [116]. This makes it particularly useful for targeting disorders of the intestine. One of the first studies to look at peppermint oil ingestion was performed in 1984 [117] in which a comparison was done between the oral administration of gelatine capsules containing peppermint oil versus a *Colpermin* preparation (Tillotts Laboratories), a commercial peppermint preparation. The comparison was done in six healthy volunteers and repeated in six ileostomy subjects. The authors measured the levels of menthol in urine and found that *Colpermin* led to a more delayed-release of menthol compared to a gelatine capsule [117]. A second study conducted 3 years later, compared the *Colpermin* preparation with another new capsule administration (*Mintec* SK&F Ltd.). The authors affirmed that the *Colpermin* preparation delivered menthol more effectively to the distal small intestine and ascending colon than the *Mintec* formulation [116]. In 1990, Kaffenberger [118] studied the administration of 180 mg of peppermint oil in an enteric-coated capsule, identifying menthol glucuronide in urine by GC-FID [118].

In another study focusing on the kinetics of menthol metabolism [119], the authors used ^13^C glucose administered with peppermint oil to detect ^13^C-menthol glucuronide. In a later study, the same authors used deuterated water administered with peppermint to detect menthol glucuronide by ^2^H-NMR spectroscopy [120]. Finally, the most recent study analyzed urine and plasma by GC-MS after intragastric administration of peppermint oil [121]. Approximately, 70% of the administered menthol and its metabolites were excreted in the urine, and this amount fluctuated independent of the dose. The main metabolite identified in plasma and urine was menthol glucuronide along with lesser amounts of mono-hydroxylated menthol glucuronic acid and di-hydroxylated menthol glucuronic acid.

While menthol is highly abundant in peppermint (40%), it can also be found in sunflower petals (essential oils), tarragon (0.1%) [122], basil, and thyme (traces). However, due to the high menthol content in peppermint compared to the other two herbs and given that sunflower petals are not an edible food, menthol can be considered a specific marker of peppermint. Given that menthol glucuronide is also detectable in urine, this compound could also be a suitable biomarker of peppermint intake, particularly after capsular oral administration. However, further analysis in peppermint administration is needed for a suitable evaluation of menthol as potential food intake biomarker. In the case of spearmint, because no studies have yet been performed for spearmint BFIs, it is clear that spearmint interventions studies need to be undertaken.

#### Saffron

Saffron is among the world’s most costly spices. It comes from the dried flower stigma of *Crocus sativus*. This is a flower that is native to Southwest Asia but which is now cultivated in Greece, India, Iran, Morocco, and Spain. Saffron is mainly used as a seasoning and coloring agent in food, particularly in Persian, Indian, European, and Arab cuisines. Saffron contains more than 150 volatile and aroma-yielding compounds. Safranal (2,6,6-trimethyl-1,3-cyclohexadiene-1-carboxaldehyde) is the major compound (70%) in the volatile fraction of saffron [123, 124]. Saffron also has a number of non-volatile active components many of which are carotenoids, including zeaxanthin, lycopene, and various α- and β-carotenes. However, the golden yellow-orange color of saffron is primarily the result of the carotenoid α-crocin, a glycosyl ester of crocetin. Picrocrocin (4-(β-d-glucopyranosyloxy)-2,6,6-trimethyl-1-cyclohexene-1-carboxaldehyde) has also been found in saffron spice from 0.8 to 26.6% on a dry basis. This compound is responsible for saffron’s bitter taste [124]. In addition, saffron contains two important vitamins: riboflavin and thiamine. Riboflavin values range from 56 to 138 μg/g, and are the highest reported for any food. Thiamine values range from 0.7 to 4 μ g/g, which are well within the range of values reported in many vegetables [125]. Saffron extracts and tinctures have been used as antispasmodic agents, gingival sedatives, nerve sedatives, expectorants, stimulants, and aphrodisiacs.

Only one study has explored BFIs for saffron [126]. This was a single dose study that looked at saffron products in the plasma of four healthy volunteers using solid phase extraction—high-performance liquid chromatography (SPE-HPLC). Saffron was administered in the form of saffron tea [126]. Crocetin was tracked from the plasma of subjects and was found to be rapidly absorbed, being detected in systemic circulation after 2 h of administration, with the compound still being present for up to 24 h. A separate pharmacokinetic study that used purified crocetin extracted from *Gardenia jasminoides* fruit showed a strong dose-dependent absorption profile into the bloodstream of 10 healthy adult subjects [127].

While crocetin is also present in *Gardenia jasminoides*, this particular flower is not considered a common edible food so, in this regard, crocetin could be considered a specific BFI of saffron in plasma. The analytical performance of this biomarker has been well documented. However, additional studies are needed with regard to cumulative aspects (robustness in complex meals). Likewise, a comparison with other markers for the same food could certainly help with validating this BFI.

#### Sage

Sage or *Salvia officinalis* is a medicinal plant belonging to the *Lamiaceae* family. It is an aromatic herb native to the Mediterranean region but now widely distributed throughout the world. Sage has been used in traditional medicine for the treatment of seizures, ulcers, gout, rheumatism, inflammation, dizziness, tremors, paralysis, diarrhea, and hyperglycemia [128]. Sage has a savory, slightly peppery flavor. It is strongly aromatic, and is characterized by a medicinal, lemony, or bitter taste. It is used for seasoning and flavoring in many different foods including sausages and stuffing. The major components present in sage are α-thujone (11.55–19.23%), viridiflorol (9.94–19.46%), 1,8-cineole (8.85–15.60%), camphor (5.08–15.06%), manool (5.52–13.06%), β-caryophyllene (2.63–9.24%), α-humulene (1.93–8.94%), and β-thujone (5.45–6.17%) [129]. Some of the major phenolic compounds found in sage are rosmarinic acid, caffeic acid, carnosol, and carnosic acid. All of these polyphenols are also present in rosemary, thyme, or oregano as previously discussed.

Only one BFI study has been performed on sage in humans [130]. In this acute study, a single female volunteer was involved in a pharmacokinetic evaluation of 1,8-cineole after ingestion of sage tea [130]. The compounds identified in plasma and urine by GC-MS and LC-MS were 1,8-cineole and a number of its derivatives (2-hydroxy-1,8-cineole, 3-hydroxy-1,8-cineole, 7-hydroxy-1,8-cineole, 9-hydroxy-1,8-cineole). 1,8-cineole is also known as eucalyptol, and is a major component of essential oils from *Eucalyptus polybractea*. This monoterpene is present in numerous spices, such as rosemary, sage, basil, and laurel. It is also used in pharmaceutical preparations to treat coughs, muscular pain, neurosis, rheumatism, asthma, and urinary stones [131].

As mentioned earlier, other compounds present in rosemary and thyme are also present in sage. Interestingly, there are no intervention studies reporting any of these compounds after sage intake. While 1,8-cineole is detectable in urine after sage intake, it is important to note that 1,8-cineole is found in several other herbs belonging to the *Laminacea* family. Therefore, 1,8-cineole and its derivates cannot be considered to be specific for sage intake. Furthermore, its rapid metabolism makes it a poor BFI for most members of the *Laminacea* family. We conclude that there are no useful BFIs for sage intake that have yet been discovered or reported in the literature.

#### Turmeric (curcumin)

Turmeric is a rhizomatous herbaceous perennial plant (*Curcuma longa*) belonging to the ginger family, *Zingiberaceae.* It is native to Southeast Asia. Turmeric is a key ingredient in many Asian dishes and is used mainly as a coloring agent. The most notable phytochemical components of turmeric root include compounds called curcuminoids, such as curcumin (diferuloylmethane), demethoxycurcumin (DMC), and bisdemethoxycurcumin (BDMC). Curcumin is a polyphenolic molecule that constitutes 3.14% (on average) of powdered turmeric Curcumin is what gives the spice its yellow color [132]. The rhizome oils of turmeric contain more than 40 identifiable compounds, with the major constituents being α-turmerone (30–32%), aromatic-turmerone (17–26%), and β-turmerone (15–18%) [133].

Curcumin is a particularly well-studied spice. A total of 18 research papers were found for this review just for curcumin. This interest is likely due to the multiple biological or health activities attributed to it, including antioxidant, anti-inflammatory, and anti-tumor activities [134]. Recent clinical studies with curcumin have demonstrated additional health benefits relating to treating immune deficiencies, improving cardiovascular health, treating depression [135], combating Alzheimer’s disease, treating diabetes [136], arthritis, and inflammatory bowel disease [137, 138]. Curcumin has very low oral bioavailability, which is primarily due to its poor solubility, low absorption, rapid metabolism, and systemic elimination. As a result, most ingested curcumin is excreted through the feces unmetabolized. The small portion that is absorbed is extensively converted to its water-soluble metabolites, glucuronides, and sulfates. These metabolites include curcumin-O-glucuronide (COG), curcumin-O-sulfate (COS) [25, 138], [139], [6, 140, 141], and tetrahydrocurcumin (THC) [138, 142, 143]. Apart from glucuronidation and sulfation, other biotransformations could occur, which yield other unspecific metabolites such as ferulic acid and vanillic acid [132]. COG, the major curcumin metabolite, is relatively hydrophilic compared to curcumin due to its conjugation to glucuronic acid [141].

Several pharmacokinetic studies with healthy volunteers [139, 144, 145] and colorectal cancer patients [6] revealed that curcumin has a plasma *T*_max_ between 1 and 4 h, with conjugates persisting for up to 36 h after 10 g of curcumin intake [139]. Sharma et al. [145] determined that there was no detectable curcumin or any of its metabolites in the blood or urine of patients after the administration of 440—2200 mg of “curcuma” extract per day (containing 36–180 mg of curcumin) in advanced colorectal cancer patients. Another study performed by Cheng et al. [144] demonstrated that the peak concentrations of curcumin in serum after administration of 4, 6, and 8 g of curcumin (given in the form of tablets) were 0.51, 0.64, and 1.77 μM, respectively. However, doses below 4 g were barely detectable. Lao et al. [146] could not detect curcumin in the serum of volunteers with doses ranging from 0.5 to 8.0 g of curcumin.

Due to curcumin’s poor absorption, poor bioavailability, and fast excretion, several studies have explored different vehicles for curcumin administration to increase its bioavailability. Oral administration of curcumin in “C3 complex,” which consists of 450 mg of curcumin, 30 mg of demethoxycurcumin (DMC), and 20 mg of bisdemethoxycurcumin (BDMC) [6] was extensively tested by several research groups [6, 139, 141, 143, 145–147]. Nearly all groups were able to detect COG up to 24 h after oral administration of 4 g of curcumin. However, no curcumin could be detected for the same subjects after the same time period [141]. This suggests that curcumin-O-glucuronide (COG) has a much higher half-life and is more suitable as a curcumin/turmeric BFI.

Another interesting study of curcumin bioavailability has been conducted by Jager et al. [138]. These researchers studied the absorption of formulated curcumin versus unformulated curcumin. Their formulated curcumin used a combination of a hydrophilic carrier, cellulosic derivatives, and natural antioxidants. This formulation was found to significantly increase curcuminoid levels in the blood in comparison to the unformulated product [138]. Theracurmin, a colloidal nanoparticle dispersion of curcumin, is reported to have a much higher bioavailability (27-fold higher) than any other available preparations [148]. A recent pharmacokinetic study conducted in both mice and humans [149], looked at fresh turmeric-derived curcuminoids versus dry turmeric-derived curcuminoids. These researchers showed that fresh curcuminoids permitted better plasma delivery and better absorption. Baum and co-workers suggested that curcumin consumed with food appears to accelerate its absorption [132].

Almost all of turmeric/curcumin intake studies that we found were performed in blood (plasma or serum). A smaller number of studies looked at other biofluids or excreta such as urine and feces. In almost all cases, curcumin was barely detected. Moderately suitable biomarkers of curcumin consumption were reported in plasma after the intake of formulated curcumin, such as BDMC, DMC, THC, COG, and COS, as opposed to unformulated administration. However, in feces and colon tissue, only curcumin itself could be detected. In conclusion, formulated curcumin, curcumin biotransformation products, and curcuminoids can be detected in plasma and in feces, but due to the fact that curcumin was administered in a formulated preparation, these biomarkers cannot be considered robust BFIs of turmeric/curcumin consumption.

## Conclusion

As far as we are aware, this is the first comprehensive review of biomarkers of food intake for common herbs and spices. We believe it provides a useful overview of what is known, how most herb and spice BFI studies are done, and what needs to be done to improve the current state of knowledge of herb and spice BFIs. Based on our analysis, we found that most BFI studies of herbs and spices were typically performed through the administration of capsules, tablets or other “artificial” forms. In many cases, these studies administered a particularly abundant, carefully purified chemical component of the herb or spice, not in the form of the whole food. Technically, this means that this particular compound or family of compounds was/were evaluated in a supervised manner, not in an untargeted manner using the whole food (spice or herb) of interest. This suggests that potentially synergistic or antagonistic factors concerning the consumption of the herb or spice along with possible matrix effects in terms of absorption and bioavailability were not typically evaluated in these studies. Given that herbs and spices are normally consumed in combination with other foods, the influence of other food constituents taken in the same meal was not particularly well addressed in any of the studies that we reviewed. Because our focus was on human metabolism and biomarkers of human consumption, we restricted our reviews to human intervention trials. For the most part, animal models were not considered, except for a few specific cases where interesting or compelling biomarkers was reported. Overall, we were surprised by how little work has been done on BFIs in herbs and spices. From an initial list of 25 herbs and spices, we found that only 6 had useful or sufficiently robust BFI studies, and in many cases only a single study was completed. Relatively few fully validated BFIs were identified, although several promising or putative BFIs were described, and these will likely be confirmed if further validation studies are completed. Based on our data, it is clear that further research needs to be performed in the evaluation of herbs and spices in human intervention studies.

## Additional file

Additional file 1: **Tables S1.** and **Table S2.** describing the literature search criteria for herbs and spices. (DOCX 17 kb)
