# Supplementary material for: Herbs and Spices- Biomarkers of Intake Based on Human Intervention Studies – A Systematic Review
Source: Genes Nutr. 2019 May 22;14:18. doi: 10.1186/s12263-019-0636-8 (PMC6532192; doi:10.1186/s12263-019-0636-8)
Supplement: Supplementary file 1 — Tables S1. and Table S2. describing the literature search criteria for herbs and spices. (DOCX 17 kb) [file 12263_2019_636_MOESM1_ESM.docx]

**Additional file 1**: Tables S1 and S2 describing the literature search criteria for herbs and spices.

**Table S1.** Common keywords.

| **Operator** | **Database** | **Field** | **Keywords** |
| --- | --- | --- | --- |
|  | ***Pubmed*** | *All Fields* | biomarker* OR marker* OR metabolite* OR biokinetics OR biotransformation |
|  | ***Web of Science*** | *Topic* |  |
|  | ***Scopus*** | *Article Title/ Abstract/ Keywords* |  |
| **AND** | ***Pubmed*** | *All Fields* | trial OR experiment OR study OR intervention |
|  | ***Web of Science*** | *Topic* |  |
|  | ***Scopus*** | *Article Title/ Abstract/ Keywords* |  |
| **AND** | ***Pubmed*** | *All Fields* | human* OR men OR women OR patient* OR volunteer* OR participant* |
|  | ***Web of Science*** | *Topic* |  |
|  | ***Scopus*** | *Article Title/ Abstract/ Keywords* |  |
| **AND** | ***Pubmed*** | *All Fields* | urine OR plasma OR serum OR blood OR excretion |
|  | ***Web of Science*** | *Topic* |  |
|  | ***Scopus*** | *Article Title/ Abstract/ Keywords* |  |
| **AND** | ***Pubmed*** | *All Fields* | intake OR meal OR diet OR ingestion OR consumption OR eating OR drink* OR administration |
|  | ***Web of Science*** | *Topic* |  |
|  | ***Scopus*** | *Article Title/ Abstract/ Keywords* |  |

**Table S2.** Specific keywords for herbs and spices.

| **Operator** | **Database** | **Field** | **Keywords** |
| --- | --- | --- | --- |
| **AND** | ***Pubmed*** | *All Fields* | “anise” OR “Pimpinella anisum” OR “basil” OR “Ocimum basilicum” OR “black pepper” OR “Piper nigrum” OR “caraway” OR “Carum carvi” OR “chilli pepper” OR “Capsicum annuum” OR “Capsicum baccatum” OR “Capsicum chinense” OR “Capsicum frutescens” OR “Capsicum pubescens” OR “cinnamon” OR “Cinnamomum” OR “clove” OR “Syzygium aromaticum” OR “cumin” OR “Cuminum cyminum” OR “turmeric” OR “Curcuma longa” OR “dill” OR “Anethum graveolens” OR “fennel” OR “Foeniculum vulgare” OR “fenugreek” OR “Trigonella foenum-graecum” OR “ginger” OR “Zingiber officinale” OR “lemongrass” OR “Cymbopogon” OR “marjoram” OR “Origanum majorana” OR “nutmeg” OR “Myristica fragrans” OR “oregano” OR “Origanum vulgare” OR “parsley” OR “Petroselinum crispum” OR “peppermint” OR “Mentha x piperita” OR “rosemary” OR “Rosmarinus officinalis” OR “saffron” OR “Crocus sativus” OR “sage” OR “Salvia officinalis” OR “spearmint” OR “Mentha spicata” OR “tarragon” OR “Artemisia dracunculus” OR “thyme” OR “thymus vulgaris” |
|  | ***Web of Science*** | *Topic* |  |
|  | ***Scopus*** | *Article Title/ Abstract/ Keywords* |  |
| **NOT** | ***Pubmed*** | *All Fields* | Ginger [Author] OR Parsley[Author] OR Sage[Author] OR Dill[Author] OR Pimpinella [Author] OR Basil[Author] OR Artemisia [Author] OR Cumin[Author] OR Thyme[Author] |
|  | ***Web of Science*** | *Topic* |  |
|  | ***Scopus*** | *Article Title/ Abstract/ Keywords* |  |
